# Integrated conjugative elements drive the formation of pandemic clones of *Escherichia coli* with hybrid chromosomes

**DOI:** 10.1093/molbev/msag085

**Published:** 2026-04-07

**Authors:** Talía Berruga-Fernández, Douglas L Huseby, Oksana Koshla, Anum Shaukat, Arijana Katana, Rama Sayed, Giorgia Marino, Diarmaid Hughes

**Affiliations:** Department of Medical Biochemistry and Microbiology, Biomedical Center, Uppsala University, Uppsala, Sweden; Uppsala Antibiotic Center, Uppsala University, Uppsala, Sweden; Department of Medical Biochemistry and Microbiology, Biomedical Center, Uppsala University, Uppsala, Sweden; Department of Medical Biochemistry and Microbiology, Biomedical Center, Uppsala University, Uppsala, Sweden; Department of Medical Biochemistry and Microbiology, Biomedical Center, Uppsala University, Uppsala, Sweden; Department of Medical Biochemistry and Microbiology, Biomedical Center, Uppsala University, Uppsala, Sweden; Department of Medical Biochemistry and Microbiology, Biomedical Center, Uppsala University, Uppsala, Sweden; Department of Medical Biochemistry and Microbiology, Biomedical Center, Uppsala University, Uppsala, Sweden; Department of Medical Biochemistry and Microbiology, Biomedical Center, Uppsala University, Uppsala, Sweden; Uppsala Antibiotic Center, Uppsala University, Uppsala, Sweden

**Keywords:** bacterial evolution, hybrid chromosomes, antibiotic resistance, ICE, conjugative plasmids

## Abstract

Pathogenic multidrug-resistant bacteria with hybrid chromosomes have emerged as a significant global healthcare threat. These include the pandemic *Escherichia coli* ST1193, the product of homologous recombination events involving two phylogenetically distant strains of *E. coli*, in which mutant alleles of the widely separated genes, *gyrA* and *parC*, generating high-level fluoroquinolone resistance were acquired. The mechanisms and frequency of hybrid formation are poorly understood. We developed a robust hybrid selection procedure and applied it to 118 clinical UTI isolates of *E. coli* mixed with suitable recipient strains. Hybrids were selected from 39% of isolates. All hybrids were recombinants of donor and recipient chromosomal DNA (median length of donor DNA 367 kb), with 90% also acquiring conjugative mobile genetic elements (MGE) from the donor. We showed that individual conjugative plasmids, and integrative conjugative elements (ICE), from donors were sufficient to drive hybrid formation. These observations strongly support conjugative chromosomal DNA transfer as the major mechanism underlying hybrid formation. ICE are genome-integrated and passively propagated but when transferring to recipients they normally do so by excising and producing their own conjugation machinery. We found that ICE were responsible for the highest frequencies of hybrid chromosome formation. They could mobilize DNA around the full length of the chromosome, including the simultaneous acquisition of mutant variants of *gyrA* and *parC*, separated by ∼826 kb, generating highly fluoroquinolone-resistant bacteria in a single event. Bacterial hybrid chromosome formation driven by conjugative MGE may be an important and widespread mechanism in the emergence and evolution of high-risk bacterial pathogens.

## Introduction

Pathogenic multidrug-resistant (MDR) bacteria with hybrid chromosomes include *Escherichia coli* ST1193 (Tchesnokova et al. 2019) and ST410 (Chen et al. 2022). These acquired high-level fluoroquinolone resistance by multi-allelic homologous recombination of quinolone-resistance mutations in *gyrA* and *parC* (and *parE* in ST410), genes that are separated by almost 1 Mb on the chromosome. Another pathogenic strain, *Klebsiella pneumoniae* ST258 (Chen et al. 2014), is a carbapenemase producer with a hybrid chromosome resulting from recombination between three strains, ST11, ST442, and ST42 replacing ∼1 Mb of chromosomal DNA in the recipient. Each of these hybrid strains is MDR, virulent, and globally widespread, making them a high-risk healthcare threat (Pitout et al. 2022; Mataseje et al. 2025).

Horizontal gene transfer (HGT) between bacteria contributes significantly to bacterial evolution (Boto 2010; Arnold et al. 2022) including the spread of antimicrobial resistance and virulence determinants (Elwell and Shipley 1980; von Wintersdorff et al. 2016; Koraimann 2018). Some clinically important Gram-positive bacteria, including *Streptococcus pneumoniae* and *Neisseria* species, are naturally competent and readily take up and recombine genomic material from neighboring cells, a mechanism that generates hybrid strains with mosaic genes. This is clinically important because it is a major mechanism for generating antibiotic-resistant and vaccine-escape variants (Dowson et al. 1989; Croucher et al. 2011; Wadsworth et al. 2025). In other Gram-positive but noncompetent species, such as *Enterococcus faecium*, large chromosomal pathogenicity islands are acquired by a conjugative mechanism (Werner et al. 2013; Novais et al. 2016). *E. coli* and *K. pneumoniae* are not naturally competent, and chromosomal HGT is expected to occur by either conjugation or bacteriophage-mediated transduction. A clinically important recent example of a Gram-negative pathogen with a hybrid chromosome is the Shiga toxin-producing *E. coli* O104:H4, associated with the sequential acquisition of prophage, plasmids, and transposons. Genomic analysis suggests that different O104:H4 isolates are not derived from an outbreak strain but instead reflect multiple events generating a similar pathogenic phenotype (Baquero and Tobes 2013; Grad et al. 2013), in contrast to the chromosomal hybrid strains ST1193, ST410, and ST258.

The mechanism of formation of ST1193, ST410, and ST258 is currently unknown, but the lengthy segments of “foreign” DNA they have acquired suggest a conjugative mechanism (Cabezon et al. 2015; Frankel et al. 2023; Lang et al. 2025). Accordingly, a conjugative plasmid might recombine into the chromosome, forming what is known as a high frequency of recombination (Hfr) strain (Kahn 1968; Cavalli-Sforza 1992) capable of driving transfer of chromosomal DNA into a recipient strain. Hfr strains were traditionally used to map genes in the bacterial chromosome (Low 1991) but have also been used to experimentally generate interspecies hybrids (Baron et al. 1959; Rayssiguier et al. 1989; Bartke et al. 2021).

Clinical isolates of *E. coli* commonly harbor conjugative and other plasmids (Wright 2007; Toleman and Walsh 2011; Lang et al. 2025). We hypothesized that some clinical isolates become transient or stable Hfr strains capable of mobilizing chromosomal DNA. In addition, most clinical isolates harbor prophage, which, if induced, could potentially transfer bacterial DNA into recipient strains by transduction (Battaglioli et al. 2011; Colavecchio et al. 2017). We developed a robust assay capable of measuring very low-frequency events in mixed populations, with no detectable background interference, to assess the ability of unmodified clinical isolates to generate chromosomal hybrids. We determined the genetic architecture of the hybrids and investigated the genetic elements driving chromosomal DNA transfer. The great majority of hybrids acquired large segments of donor chromosomal DNA suggestive of conjugative transfer. We experimentally identified conjugative plasmids, and integrative conjugative elements (ICE), as important drivers of chromosomal hybrid formation. We also identified several examples of hybrids in which recipient chromosomal DNA was mobilized into the donor strain to create a hybrid.

## Results

### *Escherichia coli* clinical isolates frequently generate chromosomal hybrids

We screened 118 unmodified clinical isolates of UTI *E. coli* (Komp Lindgren et al. 2003; Nazir et al. 2011) as potential donors of chromosomal DNA to generate bacteria with hybrid chromosomes. Phenotypic testing showed that all of these clinical isolates could grow on a defined minimal medium and were susceptible to the antibiotic apramycin. As recipient strains, we employed *E. coli* K-12 MG1655 genetically modified to be amino acid auxotrophic, apramycin-resistant, and restriction-minus (Table S1, Supplementary Material online). We chose amino acid auxotrophy (by gene deletion) and apramycin resistance (by insertion of apramycin-modifying enzyme gene), as selective and counter-selective markers, respectively, because in preliminary experiments we could not select any spontaneous prototrophs, or apramycin-resistant mutants, after assaying multiple independent cultures (>10^10^ CFU) of recipient and donor strains under the conditions used for selection of hybrids. Accordingly, this recipient genotype allowed the selection of prototrophic chromosomal hybrids on defined minimal medium with apramycin (Materials and Methods) from very large mixed bacterial populations (>10^10^ cfu) without false positives arising. In the initial round of selections, all 118 clinical isolates were tested for ability to provide DNA to repair Δ*trpE* or Δ*hisC* mutations in the chromosome of the recipient. Selected clones were validated as hybrids by whole genome sequencing. Throughout this text the term “hybrid” refers to bacterial strains selected from these mixtures having a chromosome that includes the apramycin-resistant cassette from the recipient strain and the selected amino acid biosynthesis gene from the donor strain.

**Figure 1:**
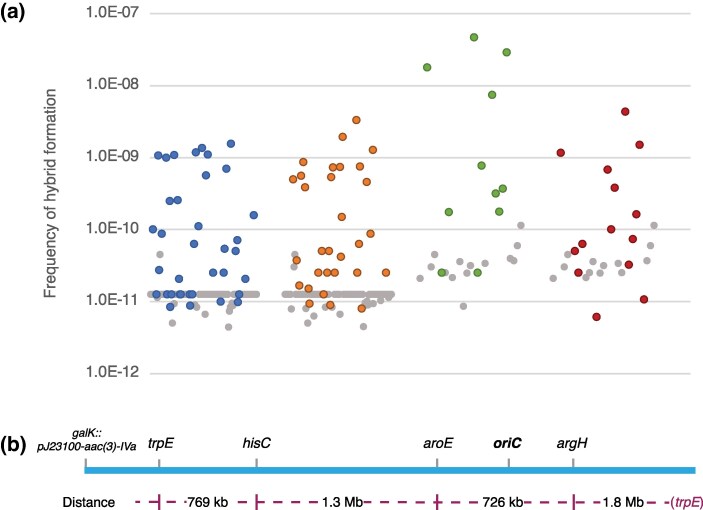
Frequencies of hybrid formation for 118 clinical isolates screened as potential donors. a) Frequency of hybrid formation per recipient cell is shown for all donors tested against the four auxotrophic *E. coli* MG1655 recipients. The selection marker *trpE* is shown in blue, *hisC* in orange, *aroE* in green, and *argH* in red. Each dot represents one donor. Gray dots represent experiments where no hybrids were isolated, that is, the limit of detection for that experiment. b) A linearized genetic map of *E. coli* MG1655 showing the relative locations of each selection marker, the location (*galK*) of the apramycin resistance cassette (aac(3)-IVa), and the distance between each marker.

Of the 118 clinical donor strains, 46 (39%) produced hybrids (Fig. 1a) with either one (37 *trpE*, 28 *hisC*) or both (21 *trpE* and *hisC*) recipients (Table S2, Supplementary Material online). To explore whether the capacity of individual donors to generate hybrids was linked to a specific region of the chromosome, a subset of 27 donor strains, representing the range of frequencies generated in the initial selections, was tested for hybrid formation with two additional recipients, with Δ*aroE* or Δ*argH* as auxotrophic markers. These genes (*trpE, hisC, aroE,* and *argH*) are located in widely spaced regions of the chromosome, separated by 726 kb to 1.8 Mb from each other (Fig. 1b). The frequencies of hybrid formation associated with individual donors spanned over three orders of magnitude, ranging from undetectable (limit of detection ∼10^−11^) up to 4.7 × 10^−8^ per recipient cell. The relative frequencies of hybrid formation as a function of the donor strain were generally consistent between the different recipients such that donors that had produced hybrids at a higher frequency in the Δ*trpE* and Δ*hisC* selections generally did so also in the Δ*aroE* and Δ*argH* selections (Table S2, Supplementary Material online).

### Genome analysis supports conjugation as the major mechanism generating hybrid strains

To facilitate analysis of hybrid genomic architecture, we made whole genome assemblies of 20 clinical isolates for use as reference genomes. Genetic and phenotypic characteristics of these 20 clinical donors belonging to 15 different multilocus sequence types (MLST) (Maiden et al. 1998), including details of their plasmids and ICE, resistance genes and alleles, and virulence genes are listed (Table S3, Supplementary Material online).

**Figure 2:**
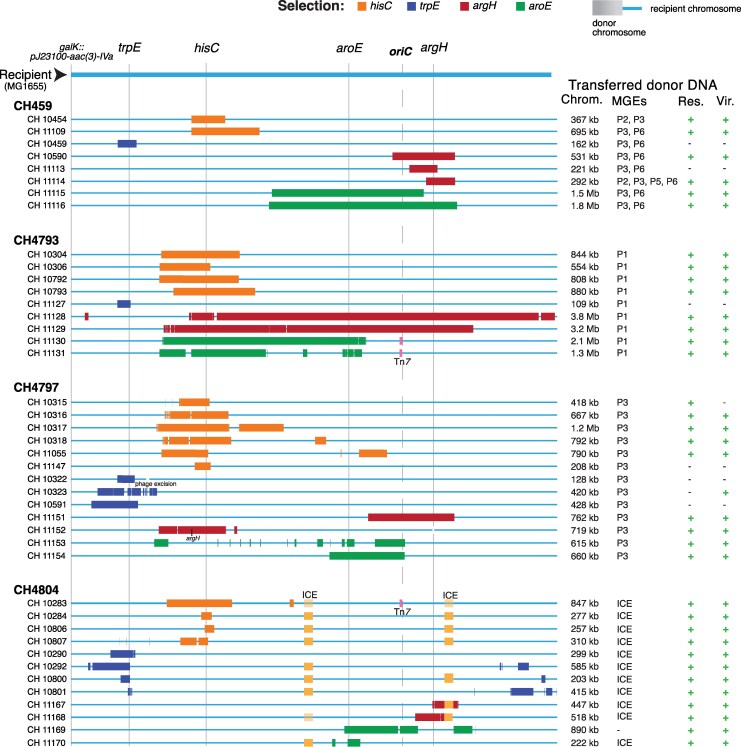
Genomic architecture of selected hybrids. The chromosomal architecture of 42 hybrids from 4 donors is shown. Recipient chromosome is shown in light blue, and acquired donor chromosomal DNA is shown in a different color depending on the selected marker. ICE (CH4804, ICE-2, Table S3, Supplementary Material online) are shown in yellow (a faded yellow color indicates <100% of the population contained the ICE); other non-conjugative donor MGEs are shown in pink. Markers in the recipient (*trpE, hisC, aroE, argH*), the location of the apramycin resistance (*galK*) and the origin of replication (*oriC*) in the recipient are indicated at the top. The total length of acquired donor chromosomal DNA, donor conjugative MGE (plasmids or ICE), acquired donor resistance and virulence genes are indicated. +/− indicates presence or absence of donor resistance or virulence genes; ICE, integrative conjugative element; *P*, plasmid; Tn, transposon.

One hundred thirty-three hybrids generated using these 20 clinical donors were whole genome sequenced and analyzed by aligning contigs to both parental reference genomes. Every selected strain had a hybrid chromosome, demonstrating the reliability and stringency of the selection procedure. We determined the size and location of every donor DNA segment recombined in each hybrid and noted resistance and virulence genes that had been acquired. Homologies at the end of each recombined segment ranged from as little as 2 bp up to 15 kb in length. Very short homologies (<10 bp) have previously been observed at junctions in bacterial chromosomal hybrids generated by Hfr-driven conjugation (Bartke et al. 2021, 2022). Hybrid formation was also reflected in changes in their MLST classification (Maiden et al. 1998) relative to their parental strains. 47/133 hybrids had a changed MLST code, with 26 of these becoming novel MLST classes (Table S4, Supplementary Material online). We also noted each plasmid and other mobile genetic elements (MGE) from a donor that were present in a hybrid (Fig. S1, Supplementary Material online). Of the 133 hybrids analyzed, 120 (90%) were found to carry at least one conjugative MGE from the donor strain, strongly suggesting that the hybrids were products of conjugation between donor and recipient bacteria. The length of donor strain chromosomal DNA in each hybrid chromosome ranged from 6 kb up to 4.8 Mb with a median length of 367 kb (Fig. S2, Supplementary Material online). Taken together with the presence of conjugative MGE in 90% of the hybrids, the long length of acquired chromosomal DNA strongly supports conjugation as the major mechanism responsible for generating the hybrid strains. In some hybrids, chromosomally integrated MGE from the donor had recombined into the recipient chromosome independently of homologous recombination of surrounding donor chromosomal DNA (eg CH11090, IS*1*; CH10403, prophage; CH11130, Tn*7*; CH10204, ICE), consistent with site-specific recombination of these elements associated with DNA transfer into the recipient (Fig. S1, Supplementary Material online). In 74 (56%) of the hybrids, the net result of the chromosomal replacement was an increase in chromosome size (range among all 133 hybrids was from −42 to +371 kb; mean +43 kb, SD 71.4). More than half of the hybrids (71/133) had acquired and recombined donor DNA into the recipient chromosome in multiple noncontiguous segments, with one hybrid having 52 noncontiguous segments (CH11153, Fig. 2, and Fig. S1, Supplementary Material online). This pattern, where chromosomal hybrids have acquired multiple discrete donor fragments, appears similar to the phenomenon called distributive conjugal transfer (DCT) described in various species of Mycobacteria (Gray and Derbyshire 2018; Orgeur et al. 2024).

The genome architectures of 42 hybrids from 4 different donors (CH459, CH4793, CH4797, and CH4804), representing some of the variation observed, are shown in Fig. 2. Genome architectures of all 133 sequenced hybrids are shown in Fig. S1, Supplementary Material online. With only one exception, each hybrid had acquired the chromosomal region carrying the wild-type copy of the selected gene (*trpE, hisC, aroE,* or *argH*) from the clinical donor. In the single exception (CH11152, Fig. 2), a duplicate *argH* gene at a second chromosomal location in the donor (CH4797 has two *argH* genes) was recombined into the recipient to generate a hybrid, which retained its original Δ*argH* mutation.

**Figure 3:**
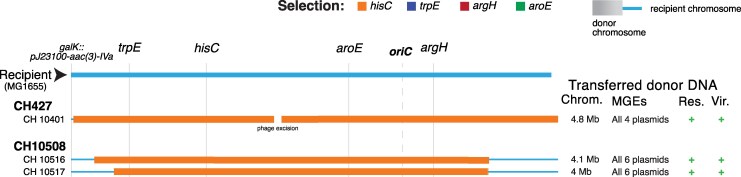
Genomic architecture of hybrids potentially formed through “reverse” conjugation. The chromosomal architecture of 3 hybrids from 2 donors is shown. All hybrids have >4 Mb of donor chromosomal DNA, as well as all donor's plasmids, including those classified as non-mobilizable. It is possible that a conjugative plasmid was transferred into the recipient, where it integrated into the chromosome and mobilized the recipient's chromosomal DNA into the clinical isolate. Recipient chromosome is shown in light blue, and acquired donor chromosomal DNA is shown in orange. For all three hybrids, the selected marker was *hisC*. Markers in the recipient (*trpE, hisC, aroE, argH*), the location of the apramycin resistance (*galK*) and the origin of replication (*oriC*) in the recipient are indicated at the top. The total length of acquired donor chromosomal DNA, donor conjugative MGE (plasmids or ICE), acquired donor resistance and virulence genes are indicated. +/− indicates the presence or absence of donor resistance or virulence genes.

Three hybrids stood out for having a distinct genetic architecture suggestive of reverse mobilization of chromosomal DNA from recipient into donor strain (Fig. 3). One, CH10401, was associated with donor CH427, while CH10516 and CH10517 were associated with donor CH10508. All three hybrids had a single large segment of donor chromosomal DNA, between 4 and 4.8 Mb in length, and, in addition, carried all of the plasmids originally present in the donor, including those classified as non-mobilizable (Table S3, Supplementary Material online). A parsimonious explanation for the formation of these hybrids is that a donor MGE transferred into the recipient strain, where it mobilized the reverse transfer of some “recipient” chromosomal DNA (including the selected apramycin-resistance region) back into the “donor” strain. After recombination, this would create a prototrophic, apramycin-resistant hybrid strain, carrying all the donor-associated plasmids.

### Putative transduction-associated hybrids

**Figure 4:**
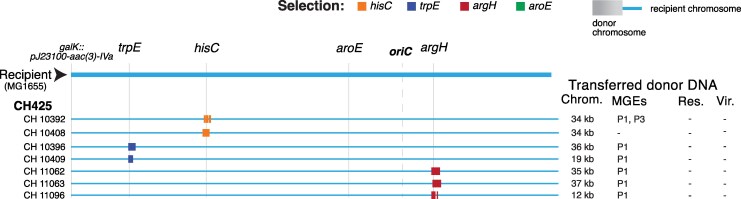
Genomic architecture of selected hybrids potentially formed through transduction. The chromosomal architecture of 7 hybrids from donor CH425 is shown. This donor has 4 prophages in its chromosome (Fig. S3, Supplementary Material online). Note that all hybrids acquired short fragments of donor chromosomal DNA. Recipient chromosome is shown in light blue, and acquired donor chromosomal DNA is shown in a different color depending on the selected marker. Markers in the recipient (*trpE, hisC, aroE, argH*), the location of the apramycin resistance (*galK*) and the origin of replication (oriC) in the recipient are indicated at the top. The total length of acquired donor chromosomal DNA, donor conjugative MGE (plasmids or ICE), acquired donor resistance and virulence genes are indicated. +/− indicates presence or absence of donor resistance or virulence genes; IS, insertion sequence; P, plasmid.

Almost every hybrid (30/31) generated with the three ST711 clinical strains as donors (CH423, CH425, and CH458) acquired a relatively short length of donor chromosomal DNA, ranging from 6 up to 44 kb (Fig. 4, and Figs S1 and S2, Supplementary Material online). The relatively short length of these acquired segments suggested the possibility that they could be produced by phage-mediated transduction. Genome analysis showed that these strains carry prophage in their chromosome (Fig. S3, Supplementary Material online). We grew overnight cultures of the donor strain CH425 in the presence of 0.04 μg/mL ciprofloxacin to induce putative prophage, pelleted the bacteria by centrifugation, filtered the supernatant through a 0.2 µM nitrocellulose filter to remove any remaining bacteria, and used the putative phage preparation to assess plaque formation ability on the auxotrophic recipient strain (Fig. S3, Supplementary Material online). The presence of plaques shows that CH425 carries an inducible prophage, but we were unable to demonstrate transduction into the auxotrophic recipient using this phage lysate. Accordingly, we have not ruled out the possibility that the “short segment hybrids” could have been generated by conjugation. Indeed, we note that 31/33 “short segment hybrids” also acquired donor strain plasmids, showing that the hybrids were involved in conjugation reactions, even if the selected segment of donor chromosomal DNA was acquired by transduction.

We also noted that 12/133 hybrids acquired a plasmid classified as non-mobilizable (Fig. S1 and Table S3, Supplementary Material online). Intriguingly, each of these 12 hybrids was generated by the same donor strains that are associated with the acquisition of short chromosomal segments (CH423, CH425, CH458, CH4790, and CH10268). This suggests the possibility that the transfer of these non-mobilizable plasmids into the recipient strain may also be due to donor-phage-mediated transduction. The relevant plasmids are each small (1,552, 2,951, and 6,200 bp) suggesting they could plausibly be packaged within a bacteriophage head for transduction. There have been reports of bacteriophage-mediated transfer of plasmids, including non-mobilizable plasmids, and chromosomal DNA by a variety of different bacteriophage into *E. coli* (Kenzaka et al. 2007; Sugiura et al. 2017).

### Resistance and virulence genes are acquired in hybrid strains

A total of 74 hybrids (56%) coincidentally acquired DNA segments carrying donor chromosomal genes conferring antimicrobial resistance (*bla*-TEM-1B, *dfrA1*, *aadA1*, and *sul2*), or mutant alleles of genes predicted to reduce antibiotic susceptibility (*gyrA*, *parC*, and various efflux pumps). MIC assays against relevant antibiotics showed that these hybrids had reduced susceptibility, consistent with the presence of the acquired genes or alleles (Table 1). Among these hybrids, selections for repair of Δ*argH* or Δ*aroE* using the clinical donor CH4793 (Fig. 2, Fig. S1, and Table S3, Supplementary Material online) generated several hybrids that acquired very long total lengths of donor DNA (2.1, 3.2, and 3.8 Mb) in 4, 23, and 41 segments, respectively, that included the quinolone-resistance-associated mutant alleles of *gyrA* and *parC* (Huseby et al. 2017; Garoff et al. 2020). These three hybrids were tested for ciprofloxacin resistance, and each had an MIC of 16 mg/L, a 1,000-fold increase above the MIC of the parental recipient strain (Table 1). These observations can potentially explain the genetic origins of hybrid clinical isolates such as *E. coli* ST1193 (Tchesnokova et al. 2019) and ST410 (Chen et al. 2022), each of which became fluoroquinolone-resistant by multi-allelic homologous recombination of quinolone-resistance mutations in *gyrA* and *parC*.

**Table 1:** MIC of hybrids that acquired antibiotic-resistance determinants from clinical strains.

Resistance alleles^a^	# Hybrids^b^	Antibiotic^c^	MIC^d^	Fold-MIC^e^
*gyrA* S83L	7	CIP	0.25	16
*gyrA* S83L, D87N	10	CIP	0.25	16
*gyrA* S83L, D87Y	1	CIP	0.25	16
*gyrA* S83L, D87N *parC* S80I	3	CIP	16	1,000
*parC* S80I	6	CIP	0.016	1
*parC* S80I, E84A	1	CIP	0.016	1
**Resistance genes^f^**				
*aadA1*	17	STR	64	4
*blaTEM-1B*	4	AMO	256	64
*dfrA1*	17	TMP	64	128
*sul2*	2	SME	64	8

^a^Mutant alleles of *gyrA*, *parC*, associated with resistance to fluoroquinolones.

^b^Number of hybrids with each genotype tested for susceptibility (in total, 74 hybrids were tested). Note that some hybrids acquired more than one resistance determinant.

^c^CIP = ciprofloxacin, STR = streptomycin, AMO = amoxicillin, TMP = trimethoprim, SME = sulfamethoxazole.

^d^MIC, the median minimal inhibitory concentration based on at least three independent measurements.

^e^Fold-MIC, fold increase above the MIC of the susceptible recipient strain without any acquired resistance determinants.

^f^Known antibiotic resistance genes acquired by the hybrid strain from the clinical donor strain.

Annotated virulence-associated genes were also coincidentally acquired by 63 (47%) of the hybrids (Fig. S1, Supplementary Material online). These included genes involved in adhesion and biofilm formation (*csgA,* mutations in *ydhQ, lpfA, hra,* and *capU*), siderophore receptors and iron transport genes (*fyuA, sitA,* and *irp2*), and hemolysins (*hylE)*.

### Individual donor plasmids or ICE are sufficient to drive hybrid formation

We noted earlier that 90% of the 133 hybrids acquired at least one conjugative MGE from the clinical donor strains. These included conjugative plasmids (106/133 hybrids) and ICE (Roche et al. 2010) integrated into tRNA-PheV and/or tRNA-PheU in 20/133 hybrids. We asked which, if any, of these conjugative elements are individually capable of promoting hybrid formation. To address this, we constructed 12 CD strains (constructed donor strains) in *E. coli* MG1655, each carrying a single conjugative plasmid or ICE from a clinical donor strain, and an additional 3 CD strains carrying 2 different MGE from the clinical strains (Table S5). Among the CD strains carrying single conjugative plasmids, 8 of the plasmids were previously found to be acquired in the majority of hybrids selected with their respective clinical donors, whereas the remaining 2 plasmids (CH468 P2 and CH4796 P1) were never found in hybrid strains (Fig. S1, Supplementary Material online).

**Figure 5:**
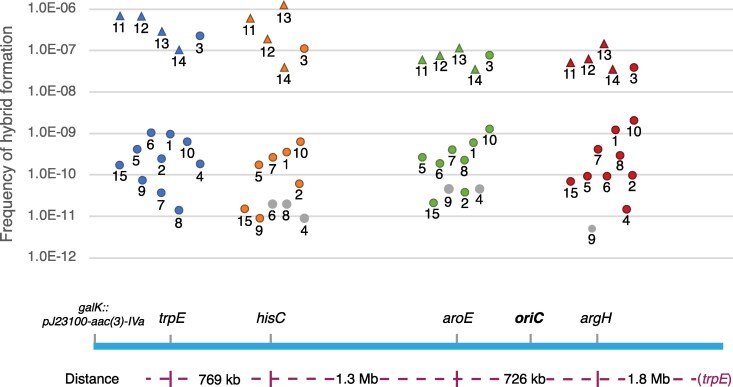
Frequency of hybrid formation driven by plasmids or ICE from clinical strains. Frequency of hybrid formation with four *E. coli* MG1655 recipients, using constructed donor strains (CD-1 to CD-15) carrying plasmids and/or ICE from clinical strains (Table S6, Supplementary Material online). Selection markers: *trpE*, blue; *hisC*, orange; *aroE*, green; *argH*, red. Each dot represents one donor. Gray dots represent experiments where no hybrids were isolated (limit of detection for that experiment). Circles represent donors with a single plasmid; triangles represent donors with ICE. The CD number of each constructed donor (Table S6, Supplementary Material online) is indicated under the relevant symbol.

Each of the strains, CD 1–15, and an isogenic control strain lacking any MGE, was tested for hybrid formation ability in mixing experiments with the four recipients previously used (*E. coli* Δ*trpE,* Δ*hisC,* Δ*aroE,* and Δ*argH*). Each of the 15 CD strains generated hybrid bacteria for at least two of the recipients, and in most cases (11/15) for all 4 recipients (Fig. 5, Table S6, Supplementary Material online). No hybrids were obtained with the isogenic donor lacking an MGE. This shows that each of the individual conjugative MGE tested (plasmid and ICE) is sufficient to drive the transfer of chromosomal DNA into a recipient to generate a hybrid. The frequency of hybrid formation between the different donors varied by over 5 orders of magnitude, from approximately 10^−11^ up to >10^−6^ (Fig. 5, Table S6, Supplementary Material online). Several results are particularly noteworthy: (i) very high frequencies of hybrid formation were obtained in each conjugation with all four donors that carried ICE (3.8 × 10^−8^ to 1.2 × 10^−6^); (ii) different conjugative plasmids originating from the same clinical strain were each found to be capable of generating hybrids; (iii) plasmid 4 from strain CH468 stood out for having a frequency of hybrid formation between 10 and 1,000 times higher than that of any of the other plasmids, including plasmid 2 from the same strain; (iv) plasmids and ICE from the same clinical strains could individually promote hybrid formation, but ICE did so at ∼1,000-fold higher frequency; (v) all 10 CD strains carrying a single conjugative plasmid, regardless of whether or not these plasmids were previously shown to be acquired in hybrids generated by clinical donors, were individually capable of generating hybrids in these experiments; (vi) and finally, in 40/51 hybrid formation experiments tested, the plasmid and/or ICE responsible for chromosomal hybrid generation was also found to have been transferred into the resulting hybrid (Table S6, Supplementary Material online). We concluded from these experiments that the ability to drive chromosomal hybrid formation is a general property of conjugative mobile elements.

### ICE can transfer widely separated regions of chromosomal DNA

Conjugative plasmids encode the ability to transfer a copy of the plasmid into a suitable recipient strain and are known to occasionally integrate into the chromosome, creating an Hfr strain that can drive transfer of chromosomal DNA (Cavalli-Sforza 1992). In contrast, ICE are chromosomally integrated elements that excise from the chromosome before conjugating a copy into a recipient strain (Toleman and Walsh 2011; Johnson and Grossman 2015; Partridge et al. 2018). However, our data (Fig. 5, Table S6, Supplementary Material online) show that ICE can also drive chromosomal DNA transfer into a recipient strain.

**Figure 6:**
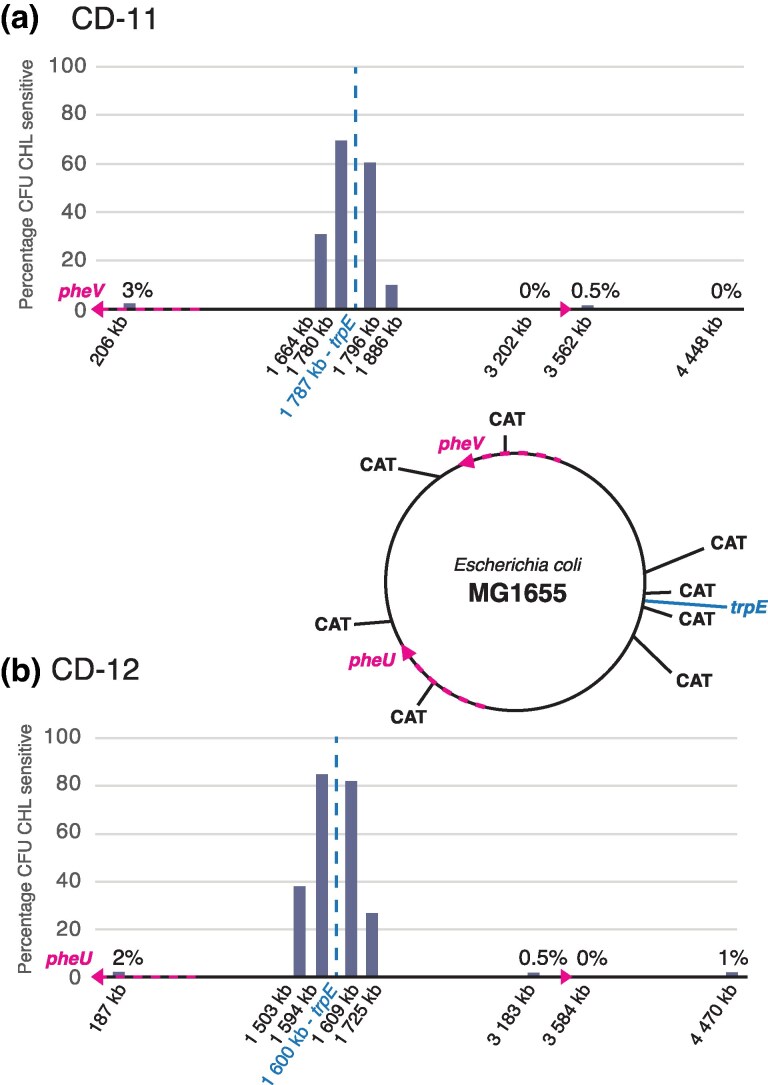
Frequency of co-transfer of unselected chromosomal regions driven by ICE. Two ICE-containing strains (CD-11 and CD-12, Table S6, Supplementary Material online) were used as donors of chromosomal DNA into MG1655, with selection for hybrids that acquired a wild-type *trpE* gene (indicated in blue) (Table S7). Panels (a) and (b): percentage of hybrids that are both *trpE^+^* and chloramphenicol-sensitive (*y* axis), relative to the distance in kb of each marker from the origin of transfer from an ICE inserted at *pheV* or *pheU* (*x* axis). Circular map: genomic map of *E. coli* K-12 MG1655 chromosome showing the relative locations of the selected marker (*trpE*) and the inserted CAT cassettes. The pink arrow indicates the location of the ICE in the donors and the direction of transfer.

We hypothesized that ICE transfers chromosomal DNA in an Hfr-like manner, beginning from its site of insertion (*pheU* or *pheV*, in these examples) and continuing processively around the chromosome. An alternative hypothesis is that ICE excises by illegitimate recombination from its site of insertion and transfers itself and adjacent chromosomal DNA, similar to the mechanism of a specialized transducing phage (Campbell 2007). However, to account for our data showing that ICE can drive hybrid formation at similar frequencies at widely separated chromosomal locations (Fig. 5), the alternative hypothesis would require the unlikely condition that ICE insert promiscuously at multiple chromosomal locations and after imprecise excision move the adjacent chromosomal region to a recipient strain. To address the hypothesis, we constructed a series of Δ*trpE* recipient strains in which a chloramphenicol acetyltransferase (CAT) gene cassette was inserted at eight different locations around the chromosome. We conducted conjugation experiments using CD-11 and CD-12 (Table S5, Supplementary Material online) in each case selecting for hybrids in which Δ*trpE* was repaired and then screening up to 200 of these selected *trp*^+^ clones for simultaneous loss of chloramphenicol resistance. The ICE in the two donor strains are integrated in inverse orientation relative to each other, and each is located >1.5 Mb away from the selected *trpE* gene (Fig. 6). Trp^+^ hybrids were obtained at frequencies ranging from 3.6 × 10^−8^ to 3.6 × 10^−7^ (Table S7, Supplementary Material online). We observed simultaneous loss of chloramphenicol resistance at sites >3 Mb away from the selected gene, with very high frequencies for locations within ±125 kb of the selected *trpE* gene (Fig. 6, Table S7, Supplementary Material online). The pattern of simultaneous acquisition or repair of distantly separated markers strongly supports that chromosomal transfer by ICE occurs in a processive Hfr-like manner. Hybrid clones that were saved and tested for the presence of ICE showed that in 5/8 clones the ICE itself had also been transferred into the hybrid (as previously observed in mixtures with clinical strains as donors, Fig. 2).

In a second experiment, to test the ability of ICE to drive the co-transfer of widely separated and clinically relevant genes, we constructed donor strains carrying ICE in the chromosome and also carrying mutant alleles of *gyrA* and *parC* associated with resistance to fluoroquinolones. These strains (CH13069, CH13071, Table 2) were used as donors in a conjugation experiment with *E. coli* Δ*hisC* (CH10162) as recipient. The distance separating *gyrA* and *parC* on the chromosome is 826 kb and mutant alleles of both genes are required to achieve high-level ciprofloxacin resistance (Garoff et al. 2020). In each experiment we selected for repair of the Δ*hisC* allele (as a positive control that ICE would drive hybrid formation as expected), and separately for acquisition of high-level ciprofloxacin resistance. The Δ*hisC* allele was repaired at frequencies >10^−7^ and ciprofloxacin resistance (simultaneous acquisition of mutant *gyrA* and *parC*) was acquired at frequencies of >10^−8^ (Table 2). In the absence of ICE, no hybrids were obtained (limit of detection ∼3 × 10^−11^). This experiment shows that the presence of ICE is sufficient to promote the creation of clinically important antibiotic-resistant hybrids through the simultaneous acquisition of widely separated mutant alleles on the bacterial chromosome.

**Table 2:** High-level ciprofloxacin resistance driven by ICE.

Donor strain	Genotype	Selection^a^	Hybrid frequency
MG1655	MG1655 WT	his^+^	<2.99E-11
		CIP	<2.99E-11
CH10369	MG1655 *gyrA* S83L, D87N; *parC* S80I + ICE in *pheV*	his^+^	7.86E-06
		CIP	5.25E-08
CH13071	MG1655 *gyrA* S83L, D87N; *parC* S80I, E84K + ICE in *malS*	his^+^	6.37E-07
		CIP	7.24E-08

^a^Selections for his^+^ were carried out on minimal media with apramycin, selections for fluoroquinolone resistance were carried out on rich media containing ciprofloxacin. Recipient strain was CH10162 (MG1655 Δ*hisC*, Table S1, Supplementary Material online).

## Discussion

Several globally widespread MDR bacterial pathogens have hybrid chromosome architectures (Chen et al. 2014; Tchesnokova et al. 2019; Chen et al. 2022), but until now, the origins, architectural variety, and potential frequency of occurrence of such strains were poorly understood. Here we have shown, using unmodified clinical isolates of *E. coli* as potential donors, that chromosomal hybrids can be generated by conjugative MGE-driven chromosomal transfer, with hybrids acquiring and recombining up to multi-megabase lengths of donor DNA, as single or multiple recombined segments. In most cases (90%) the hybrids also acquired one or more conjugative MGE (plasmids and/or ICE) from the donor strain. In addition, most hybrids simultaneously acquired chromosomal genes with adaptive traits such as resistance to antibiotics, and/or genes affecting virulence and biofilm formation (Fig. S1, Supplementary Material online). The acquisition of multiple adaptive traits during hybrid formation represents an evolutionary step-change with potential eco-evolutionary significance for the ability of hybrids to adapt and successfully exploit a wide variety of environmental niches and may be intimately linked with the global success of the pathogens referred to above.

We tested the hypothesis that individual MGE (conjugative plasmids and ICE) from clinical donor strains are capable of mobilizing chromosomal DNA into a recipient strain. Using constructed donor strains carrying individual conjugative plasmids or ICE from clinical strains, we found that all of the conjugative MGE tested could drive the formation of chromosomal hybrids, although the individual frequencies varied over five orders of magnitude. This suggests that chromosome mobilization is a promiscuous feature of conjugative MGE, and given their prevalence in natural and clinical isolates, suggests that a large fraction of *E. coli* clinical isolates will have some potential to mobilize their chromosome into a suitable recipient strain.

A striking outcome from these experiments was that ICE had a higher efficiency, relative to most plasmids tested, at generating hybrids (Fig. 5). The reason for this efficiency requires further investigation, but one simplistic explanation might be that plasmids need to integrate into the chromosome prior to mobilizing transfer, whereas ICE is already chromosomally integrated. We also showed experimentally that ICE can promote the simultaneous transfer of DNA regions around the *E. coli* chromosome, distant from its site of insertion (Fig. 6, Table S7, Supplementary Material online). This is the first study we are aware of showing that ICE from *E. coli* can mobilize chromosomal DNA to generate chromosomal hybrids. There have been only a few reports that ICE in other species (*Vibrio cholerae*, *Streptococcus algalactiae*) are capable of mobilizing chromosomal DNA (Hochhut et al. 2000; Brochet et al. 2008; Daccord et al. 2010). Because ICE are ubiquitous in the microbial world, even more so than conjugative plasmids (Guglielmini et al. 2011), their ability to mobilize chromosomal DNA may have major evolutionary implications. Antibiotic overuse and misuse will likely promote the horizontal spread of ICE, selecting directly the horizontal spread of antibiotic resistance (Botelho and Schulenburg 2021; Liu et al. 2022) and indirectly influencing the rate of formation of novel hybrid bacteria. Our data support the hypothesis that ICE are an important cause of chromosomal hybrid formation.

Another striking result from our study is that the majority of hybrids acquired multiple discrete segments of donor DNA recombined into the chromosome. This is in stark contrast to the outcome of hybrid formation mediated by Hfr strains, where whole genome sequence analysis showed that hybrids each acquired a single segment of DNA from the donor strain (Bartke et al. 2021). The acquisition of multiple segments in most hybrids, distributed widely throughout the chromosome, is similar to the phenomenon called DCT that has been observed in several mycobacterial species (Gray and Derbyshire 2018; Orgeur et al. 2024). The mechanism(s) responsible for DCT are poorly understood, but we think that the power of *E. coli* genetics can provide the possibility in future research to deepen understanding of this intriguing aspect of hybrid formation.

Our data challenge the idea that the presence of resistance genes or mutant alleles in the chromosome is not to be considered a risk for horizontal dissemination of resistance (Martinez 2014) or that only small genomic regions can be exchanged between bacteria (Boto and Martinez 2011). The existence of pathogenic and MDR hybrid clones that are globally successful (Chen et al. 2014; Tchesnokova et al. 2019; Chen et al. 2022) is in itself strong evidence for chromosomal HGT occurring at a frequency sufficient to cause healthcare problems. Our study provides insights into the broad potential for many clinical isolates to engage in chromosomal hybrid generation, and the variety of mechanisms that can underly hybrid generation. It also illustrates directly how a clinically important event such as the acquisition of mutant alleles of widely separated genes conferring high-level fluoroquinolone resistance can occur in a single event (Table 2). Indeed, we observed that more than 10% of the generated hybrids in our experiments had acquired >1 Mb of donor DNA and some had acquired >3 Mb (Fig. 2, and Figs. S1 and S2, Supplementary Material online). Intimately connected with the generation of hybrid strains is the strong possibility of switches in MLST classes, or the emergence of novel MLST classes. Reliance on MLST could result in discrepancies during surveillance of epidemics when hybrids are involved (Perez-Losada et al. 2013). An important implication of this study is that novel and potentially dangerous clinical pathogens could emerge suddenly by the generation of combinations of resistant and virulence phenotypes in a hybrid genome. Accordingly, when an infectious outbreak occurs or is suspected, the genome of the causative agent should be subjected to whole-genome sequence analysis at an early stage to determine its genetic novelty as a means of ensuring pandemic preparedness. Our study shows that potentially all, or any, of the chromosomal resistance and virulence genes of a strain could be transferred into another strain, to generate strains with novel combinations of resistance and virulence determinants, in a single genetic event.

## Materials and methods

### Bacterial strains and genetic construction

Clinical *E. coli* strains stored at Uppsala University (Komp Lindgren et al. 2003; Nazir et al. 2011) were used as potential donors of chromosomal DNA to generate bacterial hybrids. All other strains used are variants of *E. coli* K-12 MG1655 listed in Table S1. Recipient strains in conjugation experiments carried an apramycin resistance gene cassette (aac(3)-IVa) placed into *galK* behind a J23100 promoter (Anderson 2006) using lambda red recombineering with the pSIM5-Tet plasmid (Nasvall 2017). Chloramphenicol acetyltransferase gene cassettes were inserted around the chromosome using the same method. Recipient strains were made deficient in the restriction-modification system by deleting genes *mcrC, mcrB, symE, symR, hsdS, hsdM, hsdR, mrr,* and *mcrA*, and made amino acid auxotrophic by deleting either *hisC, trpE, argH*, or *aroE*. All gene deletions were made using the DIRex method (Nasvall 2017).

### Media used and growth conditions

Liquid media were either nutrient-rich LB (1% tryptone, 0.5% yeast extract, 1% NaCl) or M9 minimal media (34 mM Na_2_HPO_4_, 22 mM KH_2_PO_4_, 9 mM NaCl, 19 mM NH_4_Cl, 0.1 mM CaCl_2_, and 1 mM MgSO_4_) supplemented with 0.2% glucose. Liquid cultures were grown on a rotary shaker at 180 to 200 rpm. Solid media contained 1.5% agar (LA, LB agar; M9, minimal agar; MacConkey agar with 1% galactose). Final antibiotic concentrations in media were amikacin, 50 mg/L; apramycin, 200 mg/L; ciprofloxacin, 4 mg/L; chloramphenicol, 25 mg/L; tetracycline, 15 mg/L; trimethoprim, 16 mg/L. All cultures were incubated at 37 °C, rich media plates for 18 to 24 h, minimal media plates for 3 to 5 days.

### Filter conjugation protocol

Donor and recipient strains were independently grown overnight in antibiotic-free LB. An equal volume of donor and recipient cultures was mixed for each conjugation experiment, and 600 μL of this mixture was spread onto an 82 mm diameter, 0.2 μm pore nitrocellulose filter (Protran BA 83, Whatman, Germany) placed on antibiotic-free LA, then incubated overnight at 37 °C. Filters were removed using sterile tweezers and placed in a 50 mL Falcon tube containing 2 mL of 0.9% NaCl solution, then vortexed to resuspend all bacteria (final density 1–2 ×10^10^ CFU/mL). For selecting hybrids, 1.9 mL was aliquoted onto nine M9-apramycin plates, and the remaining 100 μL was diluted and spread on LA-apramycin and M9 plates without apramycin to calculate the CFU/mL of each parental strain. Transconjugants with hybrid chromosomes were validated by picking colonies from the M9 apramycin plates and pure-streaking them onto the same media to verify growth. To exclude the possibility of contaminants, colonies were also streaked onto MacConkey plates containing galactose, or the apramycin-resistance cassette was amplified using PCR. Genotypes were confirmed by whole genome sequencing. The frequency of hybrid formation was calculated by dividing the total number of observed hybrids by the total number of parental recipient cells. Hybrid strains were stored in LB containing 15% glycerol at −80 °C.

### Liquid conjugation protocol

Used for the isolation of conjugative MGEs from clinical isolates into *E. coli* K-12 MG1655. Donor and recipient strains were independently grown overnight in antibiotic-free LB. Then, 100 μL of each culture was pipetted into a 15 mL Falcon tube containing 1 mL of LB. This mixture was incubated 3 to 4 h at 37 °C without shaking, then plated on selective media (apramycin + amikacin, apramycin + tetracycline, or apramycin + trimethoprim), depending on the resistance genes present in the MGE. Genotypes were confirmed by whole genome sequencing.

### Transduction

Apramycin-resistance cassettes were removed (cured) from MG1655 strains by P1 phage transduction. Phage lysates were prepared as described (Thomason et al. 2007) on MG1655 but with lysate purification by filtration (0.2 μm nitrocellulose filter). For transduction, lysate (1, 10, and 100 μL) was mixed with 150 μL of overnight bacterial culture in LB with 5 μM CaCl_2_, incubated for 30 minutes at 37 °C without shaking, then plated on M9 plates with 1% galactose. After 2 to 3 days transductant colonies were picked, re-streaked on the same media, incubated 2 to 3 days, and phenotyped (galactose^+^, apramycin-sensitive). Genotypes were confirmed by whole genome sequencing.

### Polymerase chain reaction

Apramycin-resistance or CAT cassettes were amplified using Phusion High-Fidelity DNA polymerase (New England BioLabs, UK), according to the manufacturer's protocol. PCR products were purified using QIAquick PCR purification kit (Qiagen, Germany). The presence of the apramycin cassette in hybrids was verified by amplification, using Taq PCR Master Mix (Thermo Scientific, USA), with one primer having homology to the remaining portion of *galK* and the other primer to the middle of the apramycin cassette.

### Whole genome sequencing and analysis

Genomic DNA was prepared from overnight cultures using the MasterPure DNA and RNA purification kit (Epicentre, USA). Extracted DNA was suspended in milliQ water. The DNA concentration was measured using a Qubit 3.0 Fluorometer, and DNA was diluted to a final concentration of 0.2 ng/mL. For short-read whole genome sequencing, hybrid and donor DNA samples were prepared according to the Nextera XT DNA Library preparation Guide (Illumina, USA). Sequencing was performed in a MiSeq desktop sequencer, according to the manufacturer's instructions. For long-read whole genome sequencing, donor DNA samples were prepared for Nanopore sequencing following the Rapid Barcoding Sequencing Protocol (SQK-RBK004, Oxford Nanopore, UK). Sequencing was performed in a MinION Mk1B flow cell (Oxford Nanopore, UK).

Assembly of donor reference genomes was done by combining short and long read using Unicycler v0.5.0 (Wick et al. 2017). Gene annotation was added using the NCBI Prokaryotic Genome Annotation Pipeline (Tatusova et al. 2016). MLST classification of the clinical strains was done using the online tool at Pathogenwatch (https://pathogen.watch/). For identification of MGE, resistance genes, and virulence genes present in clinical strains, the online tool Mobile Element Finder v.1.0.3 (https://cge.food.dtu.dk/services/MobileElementFinder/) was used (Johansson et al. 2021). Allelic variants of genes associated with antibiotic resistance (*gyrA, parC, parE, nfsA, nfsB,* efflux pumps) were identified by aligning the genome of MG1655 to the genome of each clinical donor during hybrid analysis. For the identification of ICE, the online tool ICEfinder (https://github.com/EBI-Metagenomics/icefinder2/) was used (Liu et al. 2019). Plasmid mobility classification was carried out using Plascad (Altschul et al. 1990; Hyatt et al. 2010; Eddy 2011; Chawdry 2014; Yin et al. 2018; Che et al. 2021). The assembled reference genome for *E. coli* K-12 MG1655 was downloaded from the NCBI database (accession number: U00096.3).

Sequence data were aligned and analyzed using version 22.0.2 or later of the CLC Genomics Workbench (CLCbio, Qiagen, Denmark). Recombination junctions were defined as the first nucleotide that does not match the *E. coli* K-12 MG1655 sequence. The length of perfect homologies at either end of the transfer was determined, and this length is included in the given total fragment size (Fig. S4, Supplementary Material online).

## Supplementary Material

msag085_Supplementary_Data

## Data accessibility

The paired-end reads and assembled genomes of the strains described in the text have been deposited in the NCBI Bioprojects database, accession number PRJNA1327070. All other data discussed in the paper are available in the main text and Supplementary materials.
