# Supplementary material for: Integrated conjugative elements drive the formation of pandemic clones of Escherichia coli with hybrid chromosomes
Source: Mol Biol Evol. 2026 Apr 7;43(4):msag085. doi: 10.1093/molbev/msag085 (PMC13127887; doi:10.1093/molbev/msag085)
Supplement: msag085_Supplementary_Data [file msag085_supplementary_data.pdf]

Supplementary Information

Integrated conjugative elements drive the formation of pandemic clones of *Escherichia coli* with hybrid chromosomes

Talía Berruga-Fernández[1,2], Douglas L. Huseby[1], Oksana Koshla[1], Anum Shaukat[1], Arijana Katana[1], Rama Sayed[1], Giorgia Marino[1], Diarmaid Hughes[1,2]

[1]Department of Medical Biochemistry and Microbiology, Biomedical Center, Uppsala University, Uppsala, Sweden

[2]Uppsala Antibiotic Center, Uppsala University, Uppsala, Sweden

Corresponding author: Diarmaid Hughes, PhD

E-Mail: Diarmaid.hughes@imbim.uu.se

This PDF file includes:

Figures S1 to S4

Tables S1 to S7

Selection: ■ *hisC* ■ *trpE* ■ *argH* ■ *aroE* — donor chromosome; — recipient chromosome

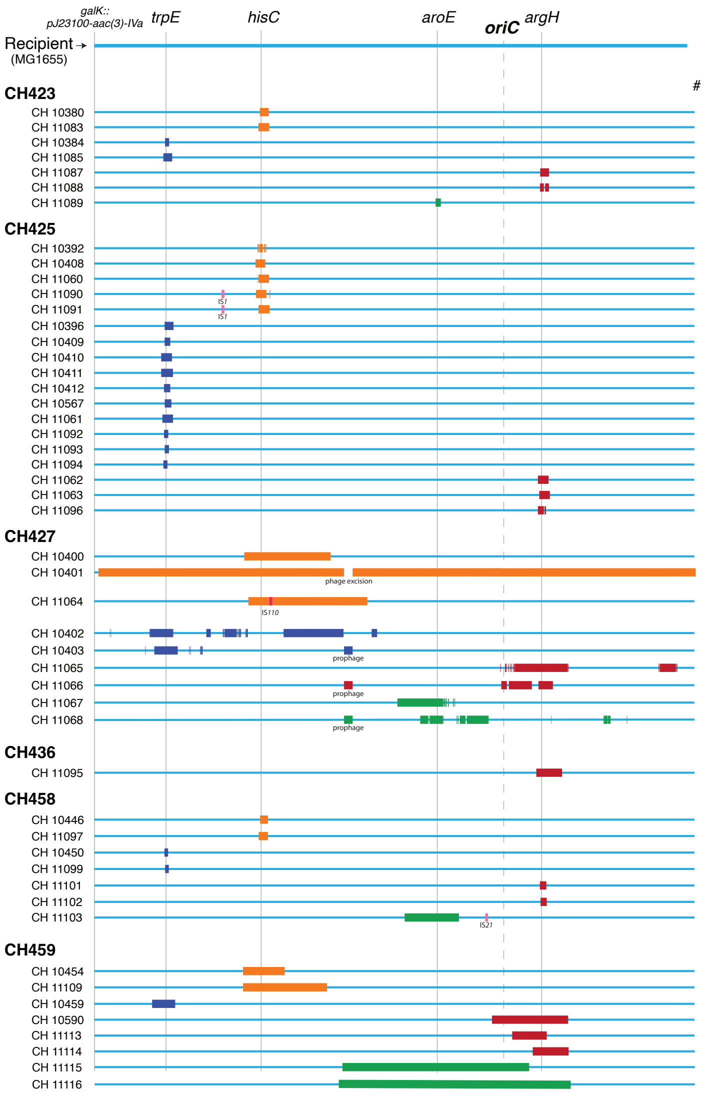

Acquired donor DNA[a]

	# Frags.	Total	Change	MGEs[b]	Resistance[c]	Virulence[d]	Other[e]
**CH423**							
CH 10380	1	43 kb	-3 kb	P1	-	-	-
CH 11083	1	44 kb	-3 kb	P1, P3	-	-	-
CH 10384	1	6 kb	-2 kb	P1, P4	-	-	-
CH 11085	1	42 kb	-2 kb	P1, P3	-	-	-
CH 11087	1	31 kb	-0.09 kb	P1	-	-	-
CH 11088	2	10 kb	-2 bp	P1, P3, P4	-	-	-
CH 11089	1	19 kb	-1 bp	P1, P3	-	-	-
**CH425**							
CH 10392	7	34 kb	-3 kb	P1, P3	-	-	-
CH 10408	1	34 kb	-3 kb	-	-	-	-
CH 11060	2	44 kb	-3 kb	P1	-	-	-
CH 11090	2	44 kb	-3 kb	P1	-	-	IS*1* inserted into *ydiN*
CH 11091	1	44 kb	-3 kb	P1	-	-	IS*1* inserted into *ydiN*
CH 10396	1	36 kb	-2 kb	P1	-	-	-
CH 10409	1	19 kb	-2 kb	P1	-	-	-
CH 10410	1	22 kb	-2 kb	-	-	-	-
CH 10411	1	37 kb	-2 kb	-	-	-	-
CH 10412	1	8 kb	-2 kb	-	-	-	-
CH 10567	1	12 kb	-2 kb	P1, P3	-	-	-
CH 11061	1	28 kb	-2 kb	P1, P3, P4	-	-	-
CH 11092	1	11 kb	-2 kb	P1, P3, P4	-	-	-
CH 11093	1	13 kb	-2 kb	P1, P3	-	-	-
CH 11094	1	14 kb	-2 kb	P1, P3	-	-	-
CH 11062	1	35 kb	-0.3 kb	P1	-	-	-
CH 11063	1	37 kb	-0.09 kb	P1	-	-	-
CH 11096	2	12 kb	-0.2 kb	P1	-	-	-
**CH427**							
CH 10400	1	613 kb	-18 kb	-	*gyrA, mdtABCD, emrK*	*terC (ygdQ)*[f]	-
CH 10401	1	4.8 Mb	+121 kb	All 4 plasmids	All	All	45 kb phage excision. 10 kb left from recipient
CH 11064	1	796 kb	-9 kb	P1	*gyrA, mdtABCD, emrK, emrA*	*terC (ygdQ)*	IS*110* inserted after *cpsG* (not present in donor)
CH 10402	25	856 kb	+17 kb	-	*gyrA, emrK*	-	-
CH 10403	9	213 kb	+17 kb	P1	-	-	45 kb prophage inserted into *ssrA*
CH 11065	18	568 kb	+3 kb	P1	*nfsB; mdtNOP*	-	-
CH 11066	4	358 kb	+61 kb	-	-	-	45 kb prophage inserted into *ssrA*
CH 11067	8	338 kb	-18 kb	-	*parC, acrF*	*terC (ygdQ)*	-
CH 11068	18	423 kb	+29 kb	-	*acrF*	-	45 kb prophage inserted into *ssrA*
**CH436**							
CH 11095	1	189 kb	+25 kb	-	-	-	-
**CH458**							
CH 10446	1	36 kb	-3 kb	P1	-	-	-
CH 11097	1	40 kb	-3 kb	P1	-	-	-
CH 10450	1	13 kb	-2 kb	P1	-	-	-
CH 11099	1	14 kb	-2 kb	P1, P3, P4	-	-	-
CH 11101	1	32 kb	-0.3 kb	P1	-	-	-
CH 11102	1	30 kb	-0.09 kb	P1, P3, P4	-	-	-
CH 11103	1	397 kb	-20 kb	P1, P3, P4	*acrF*	*terC (ygdQ)*	IS*21* inserted into *yibH*
**CH459**							
CH 10454	1	367 kb	+48 kb	P2, P3	*mdtABC*	*astA, irp2, fyuA*	-
CH 11109	1	695 kb	+33 kb	P3, P6	*gyrA, mdtABC*	*astA, irp2, fyuA*	-
CH 10459	1	162 kb	-0.4 kb	P3, P6	-	-	-
CH 10590	1	531 kb	+3 kb	P3, P6	*mdtO*	*astA*	-
CH 11113	1	221 kb	+1 kb	P3, P6	-	-	-
CH 11114	1	292 kb	+1 kb	P2, P3, P5, P6	*mdtO*	*astA*	-
CH 11115	1	1.5 Mb	+73 kb	P3, P6	*parC, parE*	*terC (ygdQ), hra, gadA*	-
CH 11116	1	1.8 Mb	+75 kb	P3, P6	*parC, parE, mdtO*	*terC (ygdQ), hra, gadA, astA*	-

20

Selection: hisC (orange) · trpE (blue) · argH (red) · aroE (green)

donor chromosome — recipient chromosome

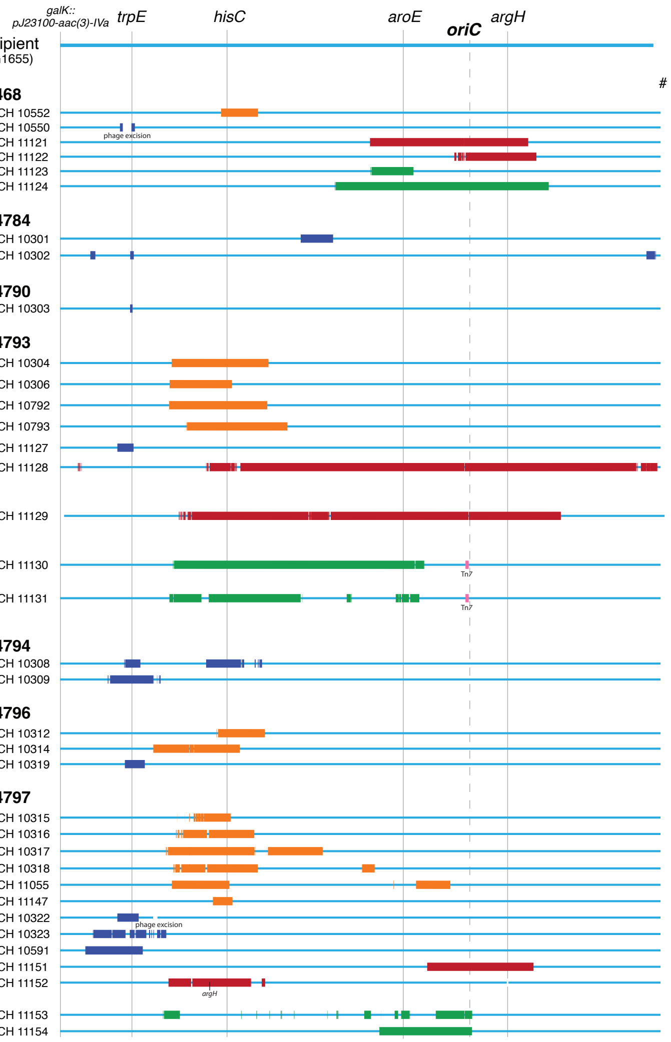

Recipient (MG1655)

Acquired donor DNA[a]

Strain	# Frags.	Total	Change	MGEs[b]	Resistance[c]	Virulence[d]	Other[e]
**CH468**							
CH 10552	1	266 kb	+19 kb	P4	*mdtABCD*	-	-
CH 10550	1	35 kb	-3 kb	P4	-	-	42 kb phage excision
CH 11121	1	1.1 Mb	-8 kb	P4	*acrF, ompR, envZ, mdtF, mdtL, mdtNOP, ytcA*	*terC (ygdQ), gadA, lpfA*	-
CH 11122	6	595 kb	+79 kb	P4	*mdtF, mdtL, mdtNOP, ytcA*	*lpfA, hra*	-
CH 11123	2	261 kb	-26 kb	P3, P4	*acrF, envZ, ompR*	-	-
CH 11124	2	1.7 Mb	+75 kb	P4	*parC, parE, acrF, envZ, ompR, mdtF, mdtL, mdtNOP, ytcA*	*terC (ygdQ), gadA, lpfA, hra*	-
**CH4784**							
CH 10301	1	283 kb	+28 kb	P1	*mdtH*	*iss*	-
CH 10302	4	118 kb	-5 kb	P1	*ompF*	-	-
**CH4790**							
CH 10303	1	12 kb	-2 kb	P1, P4, P6, P7, P9	-	-	-
**CH4793**							
CH 10304	1	844 kb	+49 kb	P1	*gyrA, mdtK, mdtABCD, bcr*	*irp2, fyuA, ydhQ*	-
CH 10306	1	554 kb	+70 kb	P1	*mdtK, mdtA*	*irp2, fyuA, ydhQ*	-
CH 10792	1	808 kb	+57 kb	P1	*gyrA, mdtK, mdtABCD, bcr*	*irp2, fyuA, ydhQ*	-
CH 10793	2	880 kb	+26 kb	P1	*gyrA, mdtK, mdtABCD, bcr, emrY*	*irp2, fyuA, ydhQ*	-
CH 11127	1	109 kb	-0.6 kb	P1	-	-	-
CH 11128	41	3.8 Mb	+371 kb	P1	*bla-TEM-1B, dfrA1, aadA1, sul2, gyrA, parC, mdtM, acrA, mdtABCD, bcr, emrK, emrY, emrA, tolC, acrEF, envZ, mdtEF, emrD, mdtL, mdtNOP, ytcA*	*pic, vat, ompT, irp2, fyuA, terC (ygdQ), hra, mchBCF, astA, chuA, gadA, ireA, lpfA*	-
CH 11129	23	3.2 Mb	+355 kb	P1	*bla-TEM-1B, dfrA1, aadA1, sul2, gyrA, parC, mdtK, mdtABCD, bcr, emrK, emrY, emrA, tolC, acrEF, envZ, mdtEF, emrD, mdtL, mdtNOP, ytcA*	*irp2, fyuA, terC (ygdQ), hra, mchBCF, astA, chuA, gadA, ireA, lpfA, ydhQ*	-
CH 11130	4	2.1 Mb	+247 kb	P1	*bla-TEM-1B, dfrA1, aadA1, gyrA, parC, mdtABCD, bcr, emrK, emrY, emrA, tolC, acrEF, envZ*	*irp2, fyuA, terC (ygdQ), mchBCF, hra, astA*	Tn7
CH 11131	16	1.3 Mb	+130 kb	P1	*dfrA1, aadA1, gyrA, mdtK, mdtABCD, bcr, emrYK, acrF, envZ*	*irp2, fyuA, hra, mchBCF, ydhQ*	Tn7
**CH4794**							
CH 10308	15	574 kb	+108 kb	P2, P4, P5	*mdtABCD*	*irp2, fyuA*	-
CH 10309	14	390 kb	+9 kb	P2, P4, P5	*sitABCD*	*iss, sitA*	-
**CH4796**							
CH 10312	3	413 kb	+34 kb	-	*gyrA, mdtABCD, bcr*	-	-
CH 10314	4	782 kb	+96 kb	P2	*marR, mdtK, mdtABCD*	*gadB, ydhQ*	-
CH 10319	2	197 kb	+32 kb	-	-	-	-
**CH4797**							
CH 10315	10	418 kb	+61 kb	P3	*mdtABC*	-	-
CH 10316	11	667 kb	+58 kb	P3	*mdtK, mdtABC*	*ydhQ*	-
CH 10317	9	1.2 Mb	+31 kb	P3	*marR, mdtK, mdtABC, emrYK, emrA*	*ydhQ*	-
CH 10318	10	792 kb	+64 kb	P3	*mdtK, mdtABC*	*terC (ygdQ), ydhQ*	-
CH 11055	4	790 kb	+63 kb	P3	*mdtK, mdtF*	*gadA, ydhQ*	-
CH 11147	1	208 kb	+68 kb	P3	-	-	-
CH 10322	1	128 kb	-42 kb	P3	-	-	23 kb phage excision
CH 10323	18	420 kb	-31 kb	P3	-	*gadB*	-
CH 10591	1	428 kb	-19 kb	P3	-	-	-
CH 11151	1	762 kb	+25 kb	P3	*mdtF, mdtL, mdtNOP, ytcA*	*gadA, lpfA*	-
CH 11152	5	719 kb	+58 kb	P3	*mdtK, mdtABC*	*ydhQ*	Donor has two *argH* genes, original deletion still present.
CH 11153	52	615 kb	+18 kb	P3	*mdtL, acrF*	*lpfA, terC (ygdQ)*	-
CH 11154	1	660 kb	-5 kb	P3	*mdtF, mdtL, acrF*	*gadA, lpfA*	-

Selection: *hisC* *trpE* *argH* *aroE* — donor chromosome — recipient chromosome

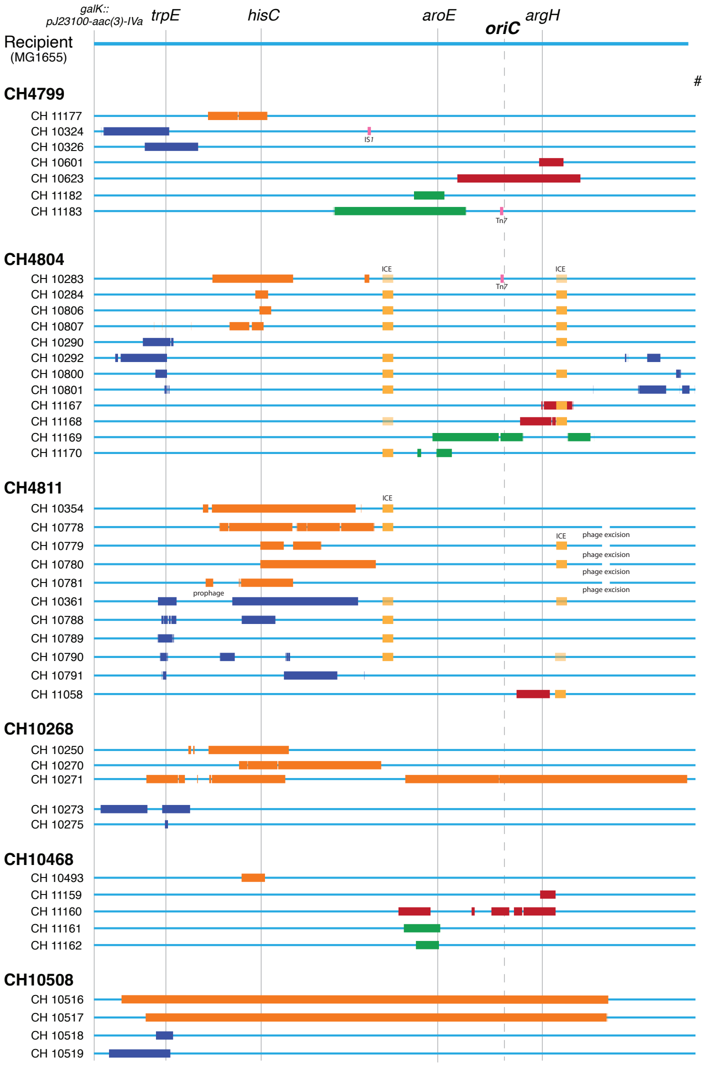

Acquired donor DNA[a]

Strain	# Frags.	Total	Change	MGEs[b]	Resistance[c]	Virulence[d]	Other[e]
**CH4799**							
CH 11177	3	501 kb	+49 kb	P2	*mdtK*	*ydhQ*	-
CH 10324	3	517 kb	-2 kb	P2	*mdfA*	-	IS*1* inserted between *iap-ygbF*
CH 10326	1	416 kb	-5 kb	P1, P2	-	*gadB*	-
CH 10601	1	184 kb	-10 kb	P2	*mdtNOP*	-	-
CH 10623	1	912 kb	+46 kb	P1, P2	*dfrA1, aadA1, mdtL, mdtNOP, ytcA, mdtF*	*lpfA, gadA*	-
CH 11182	1	176 kb	-19 kb	P2	*acrF*	-	-
CH 11183	4	1.1 Mb	+87 kb	P1, P2	*bla-TEM-1B, dfrA1, aadA1, parC, emrA, acrF*	*terC (ygdQ), astA*	Tn*7*
**CH4804**							
CH 10283	2	847 kb	+176 kb	ICE (*pheV*, *pheU*)	*dfrA1, aadA1, gyrA, mdtK, mdtABCD, bcr*	*terC (ygdQ), iha, ydhQ*	Tn*7*
CH 10284	1	277 kb	+178 kb	ICE (*pheV*, *pheU*)	*dfrA1, aadA1*	*iha*	-
CH 10806	1	257 kb	+190 kb	ICE (*pheV*, *pheU*)	*dfrA1, aadA1, mdtA*	*iha*	-
CH 10807	8	310 kb	+75 kb	ICE (*pheV*, *pheU*)	*dfrA1, aadA1*	*iha*	-
CH 10290	2	299 kb	+76 kb	ICE (*pheU*)	*dfrA1, aadA1*	*iha*	-
CH 10292	9	585 kb	+68 kb	ICE (*pheV*)	*dfrA1, aadA1, mdtH*	*iha*	-
CH 10800	3	203 kb	+90 kb	ICE (*pheV*, *pheU*)	*dfrA1, aadA1*	*iha*	-
CH 10801	14	415 kb	+109 kb	ICE (*pheV*)	*dfrA1, aadA1*	*iha*	-
CH 11167	5	447 kb	+177 kb	ICE (*pheU*)	*dfrA1, aadA1, mdtNOP*	*mhcF, hra, iha*	-
CH 11168	3	518 kb	+278 kb	ICE (*pheV*, *pheU*)	*dfrA1, aadA1, mdtNOP*	*mhcF, hra, iha*	-
CH 11169	8	890 kb	+32 kb	-	*dfrA1, aadA1, acrF, mdtF, mdtL*	*gadA, lpfA*	-
CH 11170	3	222 kb	+82 kb	ICE (*pheV*)	*dfrA1, aadA1, acrF*	*iha*	-
**CH4811**							
CH 10354	2	1.3 Mb	+106 kb	P1, ICE (*pheV*)	*gyrA, mdtK, mdtABCD, bcr, emrK, emrA*	*clbB, irp2, fyuA, yfcV, ydhQ*	20 kb prophage duplicated
CH 10778	21	1.4 Mb	+157 kb	P1, ICE (*pheV*)	*gyrA, mdtABCD, bcr, emrK, emrA*	*clbB, irp2, fyuA, yfcV, terC*	10 kb phage excision
CH 10779	3	471 kb	+77 kb	P1, ICE (*pheU*)	*mdtABCD, emrK*	*yfcV*	10 kb phage excision
CH 10780	1	983 kb	+49 kb	P1, ICE (*pheU*)	*gyrA, mdtABCD, bcr, emrK, emrA*	*yfcV, terC (ygdQ)*	10 kb phage excision
CH 10781	6	580 kb	+118 kb	P1	*gyrA, emrK*	*irp2, fyuA, clbB*	10 kb phage excision
CH 10361	2	1.3 Mb	+191 kb	P1, ICE (*pheV*, *pheU*)	*gyrA, mdtABCD, bcr, emrK, emrA*	*irp2, fyuA, clbB, yfcV*	-
CH 10788	6	478 kb	+184 kb	P1, ICE (*pheV*)	*mdtABCD*	*irp2, fyuA, clbB*	-
CH 10789	9	183 kb	+71 kb	P1, ICE (*pheV*)	-	-	-
CH 10790	11	323 kb	+133 kb	P1, ICE (*pheV*, *pheU*)	*gyrA*	-	-
CH 10791	5	438 kb	-13 kb	P1	*gyrA, emrK*	*yfcV*	-
CH 11058	1	377 kb	+102 kb	P1, ICE (*pheU*)	-	-	-
**CH10268**							
CH 10250	3	702 kb	+27 kb	P2, P3, P4	*mdtAC*	-	-
CH 10270	3	1.2 Mb	+51 kb	P2, P3, P4	*gyrA, mdtAC*	*terC (ygdQ)*	-
CH 10271	6	3.3 Mb	+151 kb	P2, P3	*parC, parE, mdtMO, mdtAC, tolC*	*hra, capU, iss, hlyE, astA, terC (ygdQ), gadA*	-
CH 10273	2	562 kb	-26 kb	P2, P3, P4	*nfsA*	-	-
CH 10275	1	20 kb	+0 kb	P2, P3, P4	-	-	-
**CH10468**							
CH 10493	1	188 kb	+5 kb	P2, P3	-	-	-
CH 11159	1	94 kb	+102 bp	P2, P3	-	-	-
CH 11160	5	644 kb	+47 kb	P2, P3	*parC, parE, mdtL, tolC*	*terC (ygdQ)*	-
CH 11161	1	341 kb	+45 kb	P2, P3	-	*terC (ygdQ)*	-
CH 11162	1	177 kb	-2 kb	P2, P3	-	-	-
**CH10508**							
CH 10516	1	4.1 Mb	+423 kb	All 6 plasmids	All except *nfsAB, acrA, mdfA*	All except *ompT, fdeC*	886 kb remaining from recipient
CH 10517	2	4 Mb	+440 kb	All 6 plasmids	All except *nfsAB, acrA, mdfA, ompF, mdtH*	All except *fdeC, ompT, csgA*	1.1 Mb remaining from recipient
CH 10518	1	142 kb	+55 kb	P1	-	-	-
CH 10519	1	553 kb	+85 kb	-	*ompF, mdtH*	*csgA, hylE*	-

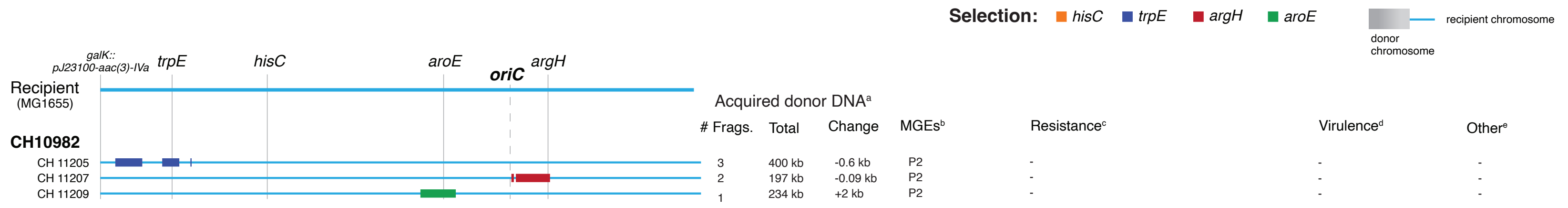

Fig. S1. Genomic architecture of 133 sequenced hybrids (clinical donors, MG1655 recipients). The chromosome architecture of 133 hybrids from 20 clinical donors is shown. Recipient chromosome is shown in light blue, and acquired regions of donor chromosome are shown in a different colours depending on the selected marker (*hisC*, orange; *trpE*, blue; *argH*, red; *aroE*, green). ICE are shown in yellow; other non-conjugative MGE acquired from the donor (IS, Tn) are shown in pink when they integrated independently (non-homologous recombination) from the rest of the donor's chromosomal DNA. A faded yellow colour indicates less than 100% of the population contained the ICE at that location. Strain numbers in bold on the left hand side refer to clinical donors, and the strain numbers for each hybrid from that donor are indicated underneath. Markers in the recipient (*trpE*, *hisC*, *aroE*, *argH*), the location of the apramycin resistance (*galK*) and the origin of replication (*oriC*) in the recipient are indicated at the top. Details on the donor chromosome transfer are indicated on the right. [a]Number of recombined fragments, total length of donor chromosome transfer, and net change in chromosome size for the recipient. [b]Plasmids and/or ICE transferred. [c]Resistance genes transferred, including variants of genes found in MG1655 (Materials and Methods). [d]Virulence genes transferred. [e]Other observations. f*terC* refers to tellurium ion resistance. (P, plasmid; ICE, integrative conjugative element; IS, insertion sequence; Tn, transposon).

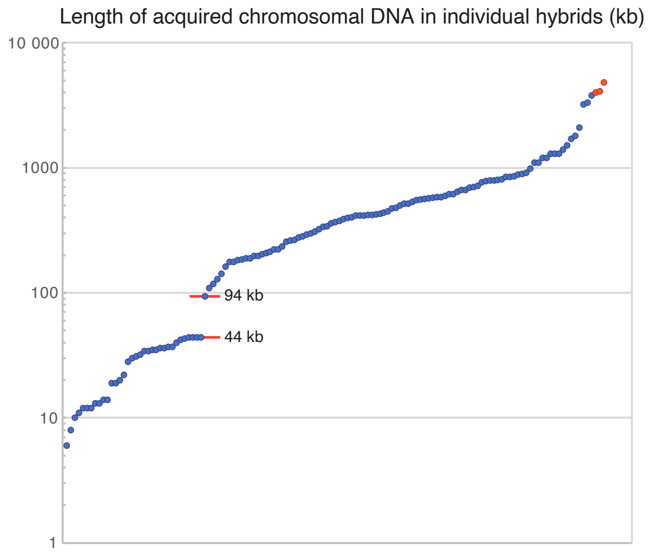

Fig. S2. Length of acquired donor chromosomal DNA. The total length of acquired DNA in all 133 hybrids is shown. Each blue dot represents one hybrid. The three orange dots represents hybrids where chromosomal DNA might have been mobilized from recipient into donor (Fig. 3). The x-axis shows DNA length in kb.

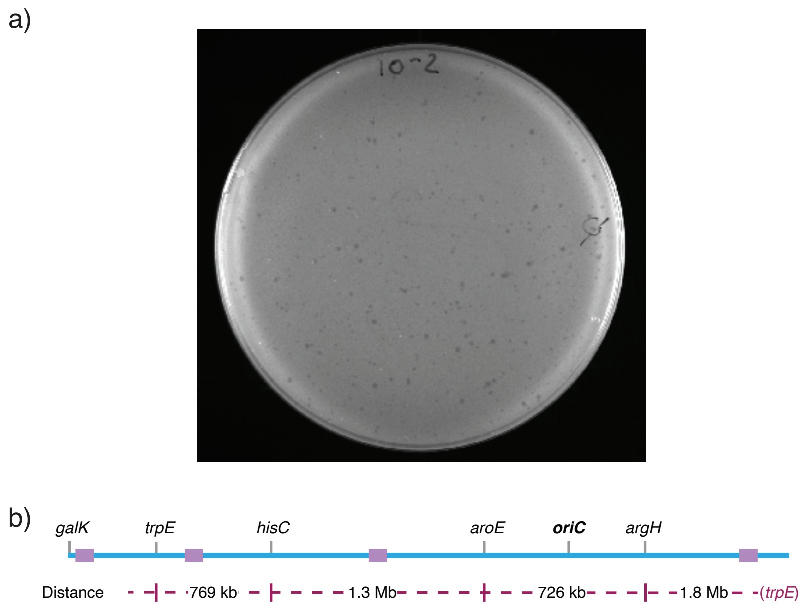

Fig S3. Prophage genomes identified in CH425. a) LB-soft-agar plate showing plaques of a phage lysate from the clinical donor CH425 grown on CH10163 (MG1655 *trpE-* recipient strain, *SI Appendix,* Table S1). **b)** A linearized genetic map of *E. coli* showing the locations of prophage genes (purple boxes) in the clinical isolate CH425 relative to the selection markers in MG1655, the location (*galK*) of the apramycin resistance cassette (aac(3)-IVa), and the distance between each marker.

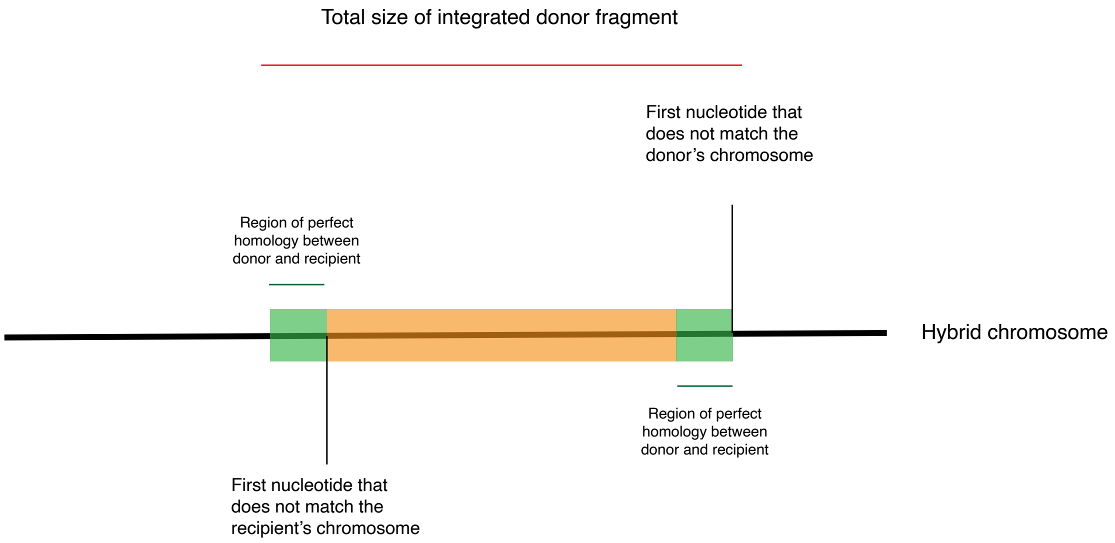

Fig. S4. Method for analysis of hybrid genomes. The contigs resulting from Illumina sequencing of the obtained transconjugants were aligned to both parental reference genomes. Recombination junctions were defined as the first nucleotide that does not match the *E. coli* K-12 MG1655 sequence (the recipient strain). The length of perfect homologies at either end of the transfer was determined and this length is included as part of the total fragment size.

Table S1. Genotypes of *E. coli* K-12 MG1655 and variants used in this study.

Strain	Genotype[a]
CH10140	Δ*mcrC-mrr*, Δ*mcrA*, *galK*::pJ23100-aac(3)-IVa
CH10162	Δ*hisC*, Δ*mcrC-mrr*, Δ*mcrA*, *galK*::pJ23100-aac(3)-IVa
CH10163	Δ*trpE*, Δ*mcrC-mrr*, Δ*mcrA*,*galK*::pJ23100-aac(3)-IVa
CH10544	Δ*argH*::del, Δ*mcrC-mrr*, Δ*mcrA*, *galK*::pJ23100-aac(3)-IVa
CH10668	Δ*aroE*::del, Δ*mcrC-mrr*, Δ*mcrA*, *galK*::pJ23100-aac(3)-IVa
CH12466	Δ*trpE, galK*::pJ23100-aac(3)-IVa, Δ*mcrC-mrr*, Δ*mcrA*, *ompW*-**CAT**-*yciE* (1796 kb from *pheV*; 1594 kb from *pheU*).
CH12467	Δ*trpE, galK*::pJ23100-aac(3)-IVa, Δ*mcrC-mrr*, Δ*mcrA*, *sohB*-**CAT**-*yciN* (1780 kb from *pheV*; 1609 kb from *pheU*).
CH12468	Δ*trpE, galK*::pJ23100-aac(3)-IVa, Δ*mcrC-mrr*, Δ*mcrA*, *ycgI*-**CAT**-*minE* (1886 kb from *pheV*; 1503 kb from *pheU*).
CH12469	Δ*trpE, galK*::pJ23100-aac(3)-IVa, Δ*mcrC-mrr*, Δ*mcrA*, *ydbL*-**CAT**-*feaR* (1664 kb from *pheV*; 1725 kb from *pheU*).
CH12470	Δ*trpE, galK*::pJ23100-aac(3)-IVa, Δ*mcrC-mrr*, Δ*mcrA*, *ygcE*-**CAT**-*queE* (206 kb from *pheV*; 3183 kb from *pheU*).
CH12471	Δ*trpE, galK*::pJ23100-aac(3)-IVa, Δ*mcrC-mrr*, Δ*mcrA*, *yhbW*-**CAT**-*mtr* (4448 kb from *pheV*; 3584 kb from *pheU*).
CH12472	Δ*trpE, galK*::pJ23100-aac(3)-IVa, Δ*mcrC-mrr*, Δ*mcrA*, *yiaZ*-**CAT**-*thiH* (3562 kb from *pheV*; 4470 kb from *pheU*).
CH12473	Δ*trpE, galK*::pJ23100-aac(3)-IVa, Δ*mcrC-mrr*, Δ*mcrA*, *fimH*-**CAT**-*gntP* (3202 kb from *pheV*; 187 kb from *pheU*).
CH10369	*gyrA* S83L, D87N; *parC* S80I; ICE in *pheV*
CH10371	*gyrA* S83L, D87N; *parC* S80I, E84K; ICE in *malS*

[a]The location of CAT (chloramphenicol acetyltransferase gene cassette) is indicated in bold letters for relevant strains. All CATs were inserted in an intergenic region and their location is indicated as gene1-CAT-gene2.

Table S2. Frequency of hybrids per recipient for all clinical donors tested.

	[a]**Conjugation frequency per recipient for each selected marker**			
**Donor**	**CH10163**	**CH10162**	**CH10668**	**CH10544**
	Δ*trpE*	Δ*hisC*	Δ*aroE*	Δ*argH*
CH407	<1.25E-11	<1.25E-11		
CH408	<1.25E-11	<1.25E-11		
CH409	**1.00E-10**	<1.25E-11		
CH410	<1.25E-11	<1.25E-11		
CH411	<1.25E-11	<1.25E-11		
CH412	<1.25E-11	<1.25E-11		
CH413	**1.25E-11**	<1.25E-11		
CH415	<1.14E-11	<7.82E-12	<2.09E-11	<2.09E-11
CH417	<1.25E-11	<1.25E-11		
CH418	**8.75E-11**	**3.75E-11**		
CH419	<1.25E-11	<1.25E-11		
CH420	<1.25E-11	<1.25E-11		
CH421	<1.25E-11	**1.67E-11**		
CH422	<1.25E-11	<1.25E-11		
CH423	**1.00E-09**	**5.58E-10**	**5.00E-11**	**2.50E-11**
CH424	**1.25E-11**	<1.25E-11		
CH425	**1.08E-09**	**8.57E-10**	**2.50E-11**	<2.05E-11
CH426	<1.25E-11	<1.25E-11		
CH427	**2.50E-10**	**3.85E-10**	**6.25E-11**	**1.75E-10**
CH428	**7.92E-12**	<7.92E-12	<2.16E-11	<2.16E-11
CH429	**1.25E-11**	<1.25E-11		
CH431	<9.26E-12	**9.26E-12**	<3.57E-11	<3.57E-11
CH432	<1.25E-11	<1.25E-11		
CH433	<1.25E-11	<1.25E-11		
CH434	<1.25E-11	<1.25E-11		
CH435	<1.25E-11	<1.25E-11		
CH436	**2.07E-11**	<6.28E-12	**6.08E-12**	<8.26E-12
CH437	**1.25E-11**	<1.25E-11		
CH438	**1.25E-11**	<1.25E-11		
CH439	<1.25E-11	<1.25E-11		
CH440	<1.25E-11	<1.25E-11		
CH441	<1.25E-11	**2.50E-11**		
CH442	<1.25E-11	<1.25E-11		
CH443	<1.25E-11	<1.25E-11		
CH444	<1.25E-11	<1.25E-11		
CH445	<1.25E-11	**5.00E-11**		

CH446	<1.25E-11	<1.25E-11		
CH447	<1.25E-11	1.25E-11		
CH448	**1.25E-11**	**5.00E-11**		
CH449	**8.31E-12**	<8.31E-12	<2.48E-11	<2.48E-11
CH450	<1.25E-11	<1.25E-11		
CH451	**1.25E-11**	**2.50E-11**		
CH452	<1.25E-11	<1.25E-11		
CH454	**6.25E-11**	**2.50E-11**		
CH455	<8.96E-12	**8.96E-12**	<3.16E-11	<3.16E-11
CH458	**1.37E-09**	**7.25E-10**	**1.00E-10**	**2.50E-11**
CH459	**1.11E-10**	**7.35E-10**	**3.80E-10**	**7.75E-10**
CH460	<1.25E-11	<1.25E-11		
CH461	<1.25E-11	<1.25E-11		
CH462	<1.25E-11	<1.25E-11		
CH463	<1.25E-11	<1.25E-11		
CH464	<1.25E-11	<1.25E-11		
CH465	<1.25E-11	<1.25E-11		
CH467	<1.25E-11	**1.50E-10**		
CH468	**1.10E-09**	**1.94E-09**	**4.32E-09**	**7.38E-09**
CH4779	<1.25E-11	<1.25E-11		
CH4780	<1.25E-11	<1.25E-11		
CH4781	<1.25E-11	<1.25E-11		
CH4782	<1.25E-11	<1.25E-11		
CH4783	<1.25E-11	<1.25E-11		
CH4784	**2.50E-11**	<1.25E-11		
CH4785	<1.25E-11	<1.25E-11		
CH4786	<1.25E-11	<1.25E-11		
CH4787	<1.25E-11	<1.25E-11		
CH4788	<1.25E-11	<1.25E-11		
CH4789	<1.25E-11	<1.25E-11		
CH4790	**1.25E-11**	<1.25E-11		
CH4791	<1.25E-11	<1.25E-11		
CH4792	<1.25E-11	<1.25E-11		
CH4793	**9.51E-12**	**3.31E-09**	**3.23E-11**	**3.17E-10**
CH4794	<1.25E-11	**2.50E-11**		
CH4795	<1.25E-11	<1.25E-11		
CH4796	**2.50E-11**	**6.25E-11**		
CH4797	**5.39E-11**	**7.44E-10**	**7.31E-11**	**1.76E-10**
CH4798	<1.25E-11	<1.25E-11		
CH4799	**6.94E-10**	**7.94E-12**	**1.63E-10**	**3.70E-10**
CH4800	<1.25E-11	<1.25E-11		

CH4801	<4.42E-12	<4.47E-12		
CH4802	<7.33E-12	<8.70E-12		
CH4803	<8.35E-12	<9.62E-12		
CH4804	**1.56E-09**	**4.56E-10**	**1.49E-09**	**2.89E-08**
CH4805	<9.40E-12	<1.00E-11		
CH4806	<8.85E-12	<8.96E-12		
CH4807	<1.25E-11	<1.25E-11		
CH4808	<1.25E-11	<1.25E-11		
CH4809	**5.00E-11**	**8.75E-11**		
CH4810	<1.25E-11	<1.25E-11		
CH4811	**7.19E-11**	**1.27E-09**	**1.07E-11**	<3.97E-11
CH4812	**9.33E-12**	<9.33E-12	<3.68E-11	<3.68E-11
CH4813	**1.25E-11**	<1.25E-11		
CH4814	<1.25E-11	<1.25E-11		
CH4815	<1.25E-11	<1.25E-11		
CH4888	<1.25E-11	<1.25E-11		
CH4889	<1.25E-11	<1.25E-11		
CH4890	<1.25E-11	<1.25E-11		
CH4891	<1.25E-11	<1.25E-11		
CH4892	**2.07E-11**	<1.03E-11	<5.95E-11	<5.95E-11
CH4893	<1.25E-11	<1.25E-11		
CH4894	<1.25E-11	<1.25E-11		
CH4895	<1.25E-11	<1.25E-11		
CH4896	<1.25E-11	<1.25E-11		
CH4897	<1.25E-11	<1.25E-11		
CH10267	<5.00E-12	<5.00E-12		
CH10268	**2.57E-10**	**1.50E-11**	<2.50E-11	
CH10343	<1.25E-11	2.50E-11		
CH10344	<1.25E-11	<1.25E-11		
CH10345	<1.25E-11	<1.25E-11		
CH10346	**1.58E-10**	<1.13E-11	<1.14E-11	<1.14E-11
CH10347	<1.25E-11	<1.25E-11		
CH10348	<1.25E-11	<1.25E-11		
CH10349	<1.25E-11	<1.25E-11		
CH10468	**1.17E-09**	**5.34E-10**	**6.79E-10**	**4.69E-08**
CH10469	<8.33E-12	<8.33E-12		
CH10507	<6.68E-12	<6.68E-12	<3.36E-11	<3.36E-11
CH10508	**5.63E-10**	**4.17E-11**	<2.50E-11	
CH10982	**1.06E-09**	**4.94E-10**	**1.16E-09**	**1.78E-08**
CH10983	**2.74E-11**	<3.03E-11	<3.03E-11	<3.03E-11
CH10984	<4.46E-11	<4.46E-11	<4.46E-11	<4.46E-11

[a]Bold type indicates hybrids were obtained (otherwise below the limit of detection for that experiment).

Table S3. Characteristics of 20 clinical donor strains sequenced

**Donor**	**ST[a]**	**Chromosomal genes**		**Plasmid and ICE/IME description**					
		**Resistance associated**	**Virulence associated**	**Number**	**Size (bp)**	**Name[b]**	**Type/insertion site[c]**	**Resistance genes**	**Virulence genes**
CH423	711	None	*gad, hlyE, lpfA, terC*[d]	Plasmid 1	103 957	IncI1	Conjugative	None	None
				Plasmid 2	78 339	IncY	Non-mobilizable	*aph(3")-Ib, aph(6)-Id, blaTEM-1B, dfrA14, qnrS1, sul2, tetA*	None
				Plasmid 3	6 231	Col (MG828)	Mobilizable	None	None
				Plasmid 4	2 951	ColpVC	Non-mobilizable	None	None
CH425	711	None	*gad, hlyE, lpfA, terC*	Plasmid 1	103 957	IncI1	Conjugative	None	None
				Plasmid 2	78 339	IncY	Non-mobilizable	*aph(3")-Ib, aph(6)-Id, blaTEM-1B, dfrA14, qnrS1, sul2, tetA*	None
				Plasmid 3	6 231	Col (MG828)	Mobilizable	None	None
				Plasmid 4	2 951	ColpVC	Non-mobilizable	None	None

CH427	3030	*blaTEM-1B, catA1, dfrB4, gyrA* S83L, D87N; *parC* S80I, E84V; *qacE, qepA4, sul1*	*gad, hlyE, lpfA, terC*	Plasmid 1	120 095	IncFIB (pB171), IncFII	Conjugative	*aac(3)-IIa, blaTEM-1B, mphA, tetB*	*traT*
				Plasmid 2	98 810	IncY	Non-mobilizable	None	None
				Plasmid 3	90 215	IncA/C2	Conjugative	None	None
				Plasmid 4	4 073	-	Mobilizable	None	None
CH436	131	*sitABCD, gyrA* S83L, D87N; *parC* S80I, E84V	*chuA, cnf1 fyuA, gad, hlyA, iha, irp2, iss, iucC, iutA, kpsE, kpsMII_K5, ompT, papC, papA_F43, sat, sitA, terC, usp, yfcV*	Plasmid 1	147 221	IncFIA, IncFIB (AP001918)	Mobilizable	*aph(3")-Ib, aph(6)-Id, blaTEM-1B, dfrA14, sitABCD, sul1, sul2*	*cma, cvaC, iucC, iutA, iroN, iss, etsC, ompT, hlyF*
				Plasmid 2	94 101	IncFII	Conjugative	*aac(3)-IIa, aac(6')-Ib-cr, blaCTX-M-15, blaOXA-1, blaTEM-1B, catB3, tetA*	*traT*
				IME	19 337	-	Between *aroA* and *ycaL*	None	None
CH458	711	None	*gad, hlyE, lpfA, terC*	Plasmid 1	103 957	IncI1	Conjugative	None	None
				Plasmid 2	78 339	IncY	Non-mobilizable	*aph(3")-Ib, aph(6)-Id, blaTEM-1B, dfrA14, qnrS1, sul2, tetA*	None

				Plasmid 3	6 231	Col (MG828)	Mobilizable	None	None
				Plasmid 4	2 951	ColpVC	Non-mobilizable	None	None
CH459	10	*blaTEM-1B, gyrA* S83L, D87N; *parC* S80I, E84V; *qepA1, sitABCD, tetB*	*astA, sitA, gad, hra, irp2, sitA, terC*	Plasmid 1	134 888	IncFIA, IncFII (pRsB107), Col156, IncFIB (AP001918)	Mobilizable	*aph(3")-Ib, aph(6)-Id, aadA5, blaTEM-1B, dfrA17, mphA, qacE, sul1, sul2, tetA*	*astA, senB, traT*
				Plasmid 2	89 298	IncFII	Conjugative	*blaTEM-35, mphA*	*traT*
				Plasmid 3	88 741	IncI1	Conjugative	*blaCTX-M-15, blaTEM-1B*	None
				Plasmid 4	36 700	IncR	Non-mobilizable	None	None
				Plasmid 5	5 165	Col156	Mobilizable	None	None
				Plasmid 6	4 072	-	Mobilizable	None	None
				Plasmid 7	1 549	-	Non-mobilizable	None	None
CH468	101	*aac(3)-IIa, aph(3")-Ib, aph(6)-Id, blaCTX-M-15, blaOXA-2, blaTEM-*	*fyuA, gad, hlyE, hra, irp2, iss, lpfA, terC, cag12*	Plasmid 1	236 000	IncHI1B (R27), IncHI1A	Conjugative	*blaCTX-M-15, blaOXA-9, aac(6')-Ib, blaTEM-1A, aadA1, aac(6')-Ib-cr*	*terC*
				Plasmid 2	165 199	IncA/C2	Conjugative	*blaCTX-M-15, msrE, armA, sul1, dfrA12, aadA2, bla-TEM-1A, qacE, blaCMY-23, mphE*	None

		1A, catA1, dfrA29, gyrA S83L, D87N, D678E; *parC* S80I; *parE* E460D; *qacE, sul1*		Plasmid 3	117 955	IncFII, IncFIA	Conjugative	None	*traT*
				Plasmid 4	108 741	Incl1	Conjugative	*dfrA1, sul2*	*cib*
				Plasmid 5	89 462	-	Non-mobilizable	None	None
				Plasmid 6	22 697	IncFIB (pB171), IncFII	Non-mobilizable	None	None
				Plasmid 7	2 302	-	Non-mobilizable	None	None
				ICE	66 224	HPI-ICEEh1	*asnT*	None	*irp2, fyuA, cag12*
				IME 1	5 230	-	*dsdX*	None	None
				IME 2	64 470	-	*yghJ*	None	None
CH4784	46	*aac(3)-IId, aadA5, dfrA17, gyrA* S83L, D87N, D678E, T611S; *parC* S80I; *parE* S458T	*fyuA, gadAB, irp2, iss, terC, cag12*	Plasmid 1	61 923	IncFII (pSE11)	Conjugative	None	*traT*
				Plasmid 2	4 082	-	Mobilizable	None	None
				ICE	58 199	ICEEcoED1a-1	*asnT*	None	*irp2, fyuA, cag12*

CH4790	23	*gyrA* S83L, D87G; *parC* S80I	*gad, hlyE, iss, lpfA, terC*	Plasmid 1	120 732	IncI1	Conjugative	*aph(3')-Ia, blaTEM-1B, dfrA14, mphA, sul2*	*cib*
				Plasmid 2	120 202	IncFIC (FII), IncFIB (AP001918)	Conjugative	*sitABCD, tetA*	*cma, cvaC, traT, iroN, sitA, iss, iucC, iutA, hylF, ompT*
				Plasmid 3	96 877	IncY	Non-mobilizable	None	None
				Plasmid 4	6 647	-	Mobilizable	None	*cea*
				Plasmid 5	4 715	-	Non-mobilizable	None	None
				Plasmid 6	4 593	ColRNAI	Mobilizable	None	None
				Plasmid 7	3 646	-	Mobilizable	None	None
				Plasmid 8	1 888	-	Non-mobilizable	None	None
				Plasmid 9	1 552	Col (MG828)	Non-mobilizable	None	None
CH4793	117	*aadA1, blaTEM-1B, dfrA1, mchB, gyrA* S83L, D87N, D678E; *parC* S80I	*astA, chuA, fyuA, gad, hra, ireA, irp2, iss, lpfA, mchB, mchC, mchF, ompT, pic, terC, vat*	Plasmid 1	98 719	IncI1	Conjugative	None	None

CH4794	131	*sitABCD, gyrA* S83L, D87Y, A828S; *parC* E84K, R455H, N459S, D475E; *parE* V136I, I529L	*chuA, fyuA, gad, ibeA, irp2, iss, kpsE, kpsMII_K5, ompT, sitA, terC, usp, yfcV*	Plasmid 1	145 611	IncFIB (AP001918), IncFII	Conjugative	*aph(6)-Id, aph(3")-Ib, sitABCD, tetB*	*cia, cvaC, traT, etsC, iutA, hlyF, sitA, iroN, iss, mchF, iucC, ompT*
				Plasmid 2	120 734	Incl1	Conjugative	*aph(3')-Ia, blaTEM-1B, dfrA14, mphA, sul2*	*cib*
				Plasmid 3	51 963	IncX1	Conjugative	*blaTEM-1B*	None
				Plasmid 4	4 100	-	Mobilizable	None	None
				Plasmid 5	3 597	-	Mobilizable	None	None
CH4796	501	*sitABCD, gyrA* S83L, D87N, D678E; *parC* S80I	*air, chuA, eilA, gad, iucC, iutA, kpsE, kpsMII_K5, lpfA, neuC, sitA, terC*	Plasmid 1	146 356	IncFII, IncFIB (AP001918)	Conjugative	*aadA1, blaTEM-1B, dfrA1, qacE, sitABCD, sul1, tetA*	*cma, cvaC, iroN, iss, sitA, traT*
				Plasmid 2	104 828	IncB/O/K/Z	Conjugative	*aph(3")-Ib, aph(6)-Id, sul2*	*traT*
				Plasmid 3	6 200	-	Non-mobilizable	*aph(3")-Ib, aph(6)-Id, sul2*	None
				Plasmid 4	1 565	Col (MG828)	Non-mobilizable	None	None
CH4797	359	*aadA5, blaTEM-1B, catA1, dfrA17, qacE, sul1, gyrA* S83L,	*gad, hra, ireA, lpfA, sitA, terC, yehABCD, hlyE, fimH, fdeC, csgA, nlpl, hha, tia, shiA*	Plasmid 1	143 813	IncFIC (FII), IncFIB (AP001918)	Conjugative	*sitABCD*	*cma, ompT, hlyF, sitA, traT, iutA, etsC, iroN, iss, iucC, tsh, anr*
				Plasmid 2	98 223	p0111	Non-mobilizable	None	None
				Plasmid 3	66 623	IncFII (pHN7A8)	Conjugative	None	*traJ, anr, traT*

		D87N; *parC* S80I		Plasmid 4	11 046	IncQ1	Mobilizable	*aph(3")-Ib, aph(6)-Id, sul2, tetA*	None
CH4799	295	*aadA1, blaTEM-1B, dfrA1, gyrA* S83L, D87N; *parC* S80I	*astA, gad, iss, lpfA, ompT, terC*	Plasmid 1	120790	IncB/O/K/Z	Conjugative	*aadA1, qacE, sul1, tetA*	*cib, traT*
				Plasmid 2	38 267	IncX1	Conjugative	None	*traT*
				Plasmid 3	6 902	-	Mobilizable	None	*cea*
				Plasmid 4	4100	-	Mobilizable	None	None
				Plasmid 5	3 597	-	Non-mobilizable	None	None
CH4804	616	*aadA1, dfrA1 gyrA* S83L, D87Y; *parC* S80I	*gad, hra, iha, iss, lpfA, mchB, mchC, mchF, ompT, terC*	Plasmid 1	159 868	IncFIC (FII), IncFIB (AP001918)	Conjugative	*aadA1, catA1, qacE, sitABCD, sul1, tetA*	*sitA, hlyF, iutA, traT, ompT, iroN, iucC, is, cma, cvaC*
				Plasmid 2	144 250	IncB/O/K/Z	Conjugative	None	*cma*
				Plasmid 3	113 030	IncFIB(pLF82)	Non-mobilizable	None	*cea*
				Plasmid 4	6 647	-	Mobilizable	*aph(3")-Ib, aph(6)-Id, sul2, tetA*	None
				ICE 1	113 351	-	*pheV*	None	*hra*
				ICE 2	99 931	-	*pheU*	*aadA1, sat2, dfrA1*	*hra, iha*
CH4811	73	*sitABCD, gyrA* S83L, D678E, A828S; *gyrB*	*cea, chuA, clbB, cnf1, focC, fyuA, gad, hra, iroN, irp2, iss, kpsE,*	Plasmid 1	35 757	-	Conjugative	None	None
				ICE	69 371	-	*pheV*	None	None

		E185D; *parC* D475E; *parE* V136I	*kpsMII_K23, mchB, mchC, mchF, mcmA, ompT, papA_F12, papC, pic, sfaD, sitA, usp, terC, vat, yfcV*						
CH10268	1284	*gyrA* S83L, D87N, D678E; *parC* S80I; *parE* S458A	*astA, capU, gad, hra, iss, terC, hlyE*	Plasmid 1	170 292	IncFIA, IncFIB (AP001918), IncFII	Conjugative	*aadA5, aac(3)-IIa, aac(6')-Ib-cr, blaCTX-M-15, blaOXA-1, catB3, dfrA17, mphA, qacE, sitABCD, sul1, tetB*	*iucC, iutA, sitA, traT*
				Plasmid 2	39 753	-	Conjugative	None	None
				Plasmid 3	31 765	IncX4	Conjugative	None	None
				Plasmid 4	6 200	-	Non-mobilizable	*aph(3")-Ib, aph(6)-Id, sul2*	None
CH10468	1284	*gyrA* S83L, D87N, D678E; *parC* S80I;	*astA, capU, gad, hra, iss, terC, hlyE*	Plasmid 1	170 290	IncFIA, IncFIB (AP001918), IncFII	Conjugative	*aadA5, aac(3)-IIa, aac(6')-Ib-cr, blaCTX-M-15, blaOXA-1, catB3, dfrA17, mphA, qacE, sitABCD, sul1, tetB*	*iucC, iutA, sitA, traT*

		parE S458A		Plasmid 2	39 753	-	Conjugative	None	None
				Plasmid 3	31 764	IncX4	Conjugative	None	None
				Plasmid 4	6 200	-	Non-mobilizable	*aph(3")-Ib, aph(6)-Id, sul2*	None
CH10508	648	*gyrA* S83L, D87N, D678E, A828S; *parC* S80I; *parE* S458A	*air, aslA, chuA, csgA, eilA, fdeC, gad, hlyE, kpsE, kpsMII_K5, lpfA, nlpI, ompT, terC, yehABCD, yfcV*	Plasmid 1	146 271	IncFIB (pB171), IncFII	Conjugative	*aac(3)-IIa, aac(6')-Ib_cr, aadA5, blaCTX-M-15, blaOXA-1, blaTEM-1B, catB3, dfrA17, ermB, mphA, qacE, sul1, tetB*	*traJ, traT*
				Plasmid 2	21 167	IncX4	Non-mobilizable	*blaTEM-35, dfrA1, fosA*	None
				Plasmid 3	4 063	.	Mobilizable	None	None
				Plasmid 4	3 005	Col440I	Mobilizable	None	None
				Plasmid 5	2 101	Col (BS512)	Non-mobilizable	None	None
				Plasmid 6	1 459	Col (MG828)	Non-mobilizable	None	None
CH10982	1284	*gyrA* S83L, D87N, D678E; *parC* S80I;	*astA, capU, gad, hlyE, hra, iss, terC*	Plasmid 1	170 330	IncFIA, IncFIB (AP001918), IncFII	Conjugative	*aadA5, aac(3)-IIa, aac(6')-Ib-cr, blaCTX-M-15, blaOXA-1, catB3, dfrA17, mphA, qacE, sitABCD, sul1, tetB*	*iucC, iutA, sitA, traT*

		parE S458A		Plasmid 2	39 753	-	Conjugative	None	None
				Plasmid 3	6 200	-	Non-mobilizable	*aph(3")-Ib, aph(6)-Id, sul2*	None

[a]ST = sequence type.

[b]Plasmid incompatibility group or name.

[c]Plasmid mobility classification or site of ICE/IME insertion in the chromosome. ICE/IME location was determined by looking at conserved genes around the ICE/IME according to MG1655.

[d]Gene *terC* refers to tellurium ion resistance protein, also known as *ygdQ, alx, yegH, tehA*. For details on databases used for classification and gene identification see Materials and Methods.

Table S4. Changes in MLST patterns of chromosomal hybrids.

Strain	Donor	Selection	DNA acquired (kb)	ST donor	ST Hybrid
CH11103	CH458	*aroE-*	396 553	711	3098
CH10401	CH427	*hisC-*	4 752 617	3030	3030
CH11067	CH427	*aroE-*	337 621	3030	7652
CH11068	CH427	*aroE-*	423 240	3030	7652
CH11121	CH468	*argH-*	1 091 813	101	*839c
CH11122	CH468	*argH-*	595 378	101	5846
CH11123	CH468	*aroE-*	261 780	101	7652
CH11124	CH468	*aroE-*	1 670 061	101	*135d
CH10304	CH4793	*hisC-*	844 400	117	*fc37
CH10306	CH4793	*hisC-*	553 655	117	*fc37
CH10792	CH4793	*hisC-*	808 260	117	*fc37
CH11128	CH4793	*argH-*	3 765 655	117	*55dc
CH11129	CH4793	*argH-*	3 162 890	117	*95fd
CH11130	CH4793	*aroE-*	2 081 843	117	2177
CH11131	CH4793	*aroE-*	1 340 574	117	*fc37
CH10309	CH4794	*trpE-*	390 699	131	1238
CH10314	CH4796	*hisC-*	781 738	501	*0a53
CH10317	CH4797	*hisC-*	1 162 518	359	*9b05
CH10318	CH4797	*hisC-*	792 371	359	1912
CH10323	CH4797	*trpE-*	419 794	359	*83db
CH10591	CH4797	*trpE-*	427 981	359	*83db
CH11055	CH4797	*hisC-*	790 692	359	1912
CH11151	CH4797	*argH-*	761 955	359	5846
CH11152	CH4797	*argH-*	718 626	359	1912
CH11153	CH4797	*aroE-*	614 641	359	*dc12

CH11154	CH4797	*aroE-*	660 360	359	*bd25
CH10324	CH4799	*trpE-*	516 836	295	34
CH10326	CH4799	*trpE-*	416 289	295	34
CH10623	CH4799	*argH-*	912 068	295	*1860
CH11177	CH4799	*hisC-*	501 326	295	178
CH11182	CH4799	*aroE-*	176 037	295	2687
CH11183	CH4799	*aroE-*	1 142 072	295	*6d3c
CH10290	CH4804	*trpE-*	299 242	616	*3023
CH10292	CH4804	*trpE-*	584 655	616	*3023
CH10801	CH4804	*trpE-*	414 509	616	48
CH11169	CH4804	*aroE-*	889 672	616	8759
CH10354	CH4811	*hisC-*	1 299 229	73	*90dd
CH10361	CH4811	*trpE-*	1 342 482	73	*ec85
CH10778	CH4811	*hisC-*	1 358 846	73	*ec85
CH10780	CH4811	*hisC-*	983 188	73	*ec85
CH10781	CH4811	*hisC-*	580 103	73	*e8d1
CH10250	CH10268	*hisC-*	702 205	1284	178
CH10270	CH10268	*hisC-*	1 202 239	1284	1488
CH10271	CH10268	*hisC-*	3 304 689	1284	*2cf8
CH10516	CH10508	*hisC-*	4 178 814	648	6870
CH10517	CH10508	*hisC-*	3 954 442	648	*dfa2
CH10519	CH10508	*trpE-*	553 026	648	*593a

Multi-locus sequence typing (MLST) of the 47 hybrids that changed from the recipient’s ST (ST10) to a new ST are shown. Novel MLST codes are indicated by a “*” character at the start of the code.

Table S5. Constructed donor strains in *E. coli* MG1655 background.

Constructed Donor Strain	Plasmids/ICE	MGE Type	Isolation method[a]	Clinical donor	Strain background[b]
CD-1	Plasmid 1	Conjugative	Selection	CH427	CH10140
CD-2	Plasmid 2	Conjugative	Selection	CH468	CH10140
CD-3	Plasmid 4	Conjugative	Curing	CH468	CH10550
CD-4	Plasmid 1	Conjugative	Curing	CH4793	CH11128
CD-5	Plasmid 1	Conjugative	Selection	CH4796	CH10140
CD-6	Plasmid 2	Conjugative	Curing	CH4796	CH10314
CD-7	Plasmid 1	Conjugative	Selection	CH4799	CH10140
CD-8	Plasmid 2	Conjugative	Curing	CH4799	CH11177
CD-9	Plasmid 1	Conjugative	Curing	CH4811	CH10791
CD-10	Plasmid 1	Conjugative	Selection	CH10508	CH10140
CD-11	ICE in *pheV*	Conjugative	Selection	CH4804	CH10140
CD-12	ICE in *pheU*	Conjugative	Selection	CH4804	CH10140
CD-13	Plasmid 1<br>ICE in *pheV*	Conjugative<br>Conjugative	Curing	CH4811	CH10778
CD-14	Plasmid 1<br>ICE in *pheU*	Conjugative<br>Conjugative	Curing	CH4811	CH10779
CD-15	Plasmid 1,<br>Plasmid 4	Conjugative<br>Mobilizable	Selection	CH4804	CH10140

[a]Constructed donor strains CD-1 to CD-15 were made by liquid conjugation between clinical donor strains and MG1655 using two different isolation methods. Selection for an antibiotic resistance gene on the plasmid/ICE of interest, then curing the recipient apramycin resistance cassette; or curing the apramycin resistance cassette from a hybrid carrying a plasmid/ICE of interest obtained from previous conjugations with clinical strains.

[b]Strain number of the recipient or hybrid used for donor construction.

Table S6. Frequency of hybrid formation for isogenic donor strains with MG1655 as recipient.

			**Hybrid frequency per recipient**							
			**MG1655 background**							
**Donor strains**	**Original donor**	**Plasmid/ ICE**	**CH10163**	**MGE transfer[a]**	**CH10162**	**MGE transfer[a]**	**CH10668**	**MGE transfer[a]**	**CH10544**	**MGE transfer[a]**
			trpE		***hisC***		***aroE***		***argH***	
MG1655	(control)	None	<3.76E-11	-	<2.99E-11	-	<4.07E-11	-	<5.15E-11	-
CD-1	CH427	P1	**9.77E-10**	18/26	**3.59E-10**	8/12	**6.10E-10**	11/15	**1.24E-09**	11/24
CD-2	CH468	P2	**2.47E-10**	0/8	**6.15E-11**	0/6	**3.82E-11**	0/2	**9.74E-11**	1/7
CD-3	CH468	P4	**2.25E-07**	80/80	**1.04E-07**	80/80	**8.08E-08**	80/80	**3.82E-08**	80/80
CD-4	CH4793	P1	**1.87E-10**	5/5	<8.87E-12	-	<4.55E-11	-	**1.50E-11**	3/3
CD-5	CH4796	P1	**4.14E-10**	11/13	**1.74E-10**	17/17	**2.67E-10**	15/15	**9.46E-11**	6/7
CD-6	CH4796	P2	**1.05E-09**	3/4	<1.94E-11	-	**1.92E-10**	1/5	**9.26E-11**	1/3
CD-7	CH4799	P1	**3.76E-11**	25/25	**2.69E-10**	9/9	**4.07E-10**	9/10	**4.12E-10**	7/8
CD-8	CH4799	P2	**1.41E-11**	N/A	<1.94E-11	-	**2.31E-10**	N/A	**2.96E-10**	N/A
CD-9	CH10508	P1	**6.39E-10**	11/17	**6.29E-10**	19/21	**1.30E-09**	30/32	**2.06E-09**	31/40
CD-10	CH4811	P1	**7.46E-11**	2/2	**8.87E-12**	1/1	<4.55E-11	-	<5.01E-12	-
CD-11	CH4804	ICE in *pheV*	**6.85E-07**	0/1	**5.96E-07**	0/1	**5.83E-08**	1/1	**5.06E-08**	1/1
CD-12	CH4804	ICE in *pheU*	**6.55E-07**	0/1	**1.87E-07**	0/1	**7.46E-08**	0/1	**6.28E-08**	1/1
CD-13	CH4811	P1, ICE in *pheV*	**2.84E-07**	2/3	**1.24E-06**	0/3	**1.15E-07**	2/3	**1.44E-07**	0/3
CD-14	CH4811	P1, ICE in *pheU*	**1.02E-07**	0/3	**3.83E-08**	2/3	**3.45E-08**	2/3	**3.45E-08**	1/3
CD-15	CH4804	P1 and P4	**1.72E-10**	5/5	**1.51E-11**	1/1	**2.12E-11**	1/1	**6.90E-11**	1/4

[a]Number of hybrid colonies positive for transfer of the MGE from the donor/number of hybrid colonies screened. N/A, not assessed.

Table S7 Frequencies of simultaneous hybrid formation at different chromosomal locations with an ICE donor.

Donor strain	CAT distance from ICE (kb)	Hybrid frequency Trp$^+$	# Trp$^+$ tested for Cam^R	Percent Trp$^+$ hybrids that lost CAT cassette
CD-11 (ICE in *pheV*)	+206	7.46E-08	200	3%
	+1 664	2.98E-07	100	31%
	+1 780	1.25E-07	100	70%
	+1 796	2.58E-07	100	61%
	+1 886	9.28E-08	100	10%
	+3 202	9.55E-08	195	0%
	+3 562	6.46E-08	198	0.5%
	+4 448	7.27E-08	197	0%
CD-12 (ICE in *pheU*)	+187	8.44E-08	200	2%
	+1 503	2.67E-07	104	38%
	+1 594	3.61E-07	104	85%
	+1 609	1.71E-07	104	82%
	+1 725	2.93E-07	100	27%
	+3 183	5.32E-08	197	0.5%
	+3 584	3.58E-08	199	0%
	+4 470	1.19E-07	200	1%

Hybrid frequencies are per recipient and percentage of colonies that lost the chloramphenicol resistance cassette (CAT) in conjugations using CD-11 and CD-12 with recipients containing CAT at different locations across the chromosome. Location of *trpE* is +1 787 kb relative to ICE in *pheV*, +1 600 kb relative to ICE in *pheU*.
